# In utero exposure to butyl benzyl phthalate induces modifications in the morphology and the gene expression profile of the mammary gland: an experimental study in rats

**DOI:** 10.1186/1476-069X-10-5

**Published:** 2011-01-17

**Authors:** Raquel Moral, Julia Santucci-Pereira, Richard Wang, Irma H Russo, Coral A Lamartiniere, Jose Russo

**Affiliations:** 1 Breast Cancer Research Laboratory, Fox Chase Cancer Center, Philadelphia, PA 19111, USA; 2 Department of Cell Biology, Physiology and Immunology, Medicine School, Universitat Autònoma de Barcelona, Bellaterra, Barcelona, 08193, Spain; 3 Department of Pharmacology and Toxicology, University of Alabama at Birmingham, Birmingham, AL 35294, USA

## Abstract

**Background:**

Environmental estrogens are exogenous estrogen-mimicking compounds that can interfere with endogenous endocrine systems. Several of these endocrine disruptors have been shown to alter normal development and influence tumorigenesis in experimental models. N-butyl benzyl phthalate (BBP), a widely used plasticizer, is a well-known endocrine disruptor. The aim of this study was to elucidate the effect of prenatal exposure to BBP on the morphology, proliferative index, and genomic signature of the rat mammary gland at different ages.

**Methods:**

*In utero *exposure was performed by gavage of pregnant Sprague Dawley CD rats with 120mg or 500mg BBP/kg/day from day 10 post-conception to delivery. Female litters were euthanized at 21, 35, 50 and 100 days. The morphology and proliferative index of the mammary gland were studied from whole mount preparations and BrdU incorporation, respectively. Gene expression profile was assessed by microarrays. Several genes found differentially expressed and related to different functional categories were further validated by real time RT-PCR.

**Results:**

Prenatal exposure of BBP induced delayed vaginal opening and changes in the post-natal mammary gland long after the end of the treatment, mainly by 35 days of age. Exposure to the high dose resulted in modifications in architecture and proliferative index of the mammary gland, mostly affecting the undifferentiated terminal end buds. Moreover, the expression profiles of this gland in the exposed rats were modified in a dose-dependent fashion. Analysis of functional categories showed that modified genes were related to immune function, cell signaling, proliferation and differentiation, or metabolism.

**Conclusions:**

Our data suggest that *in utero *exposure to BBP induced a delayed pubertal onset and modified morphology of the mammary gland. These alterations were accompanied by modifications in gene expression previously associated with an increased susceptibility to carcinogenesis.

## Background

Phthalates are widely used plasticizers that have generated growing concern for their potential adverse effect on human health. Compounds such as butyl benzyl phthalate (BBP), commonly used in vinyl tile among other products, have been shown to be potential toxicants in rats [1]. *In vivo *models, especially involving rats, have proven to be a good tool for investigating the effect of environmental compounds on health, and thus obtaining evidence of the potential risk of such compounds on humans. Sprague-Dawley rats are widely used in toxicological and risk assessment studies due to the fact that they have a metabolism similar to humans [2]. Perinatal exposure of rats to BBP and its major metabolite, monobenzyl phthalate, has been shown to have an antiandrogenic effect, inducing significant alterations in the reproductive system of male offspring. A few published studies have also observed the effects of BBP in females, such as alterations in ovarian weight and anogenital distance [3], or modifications in the progesterone receptor expression in the preoptic area [4].

Although the risks of BBP to human health are controversial, the European Commission has banned BBP from toys and child care items because of its reproductive toxicity [5]. BBP is one of the phthalates studied by the National Toxicology Program (NTP) Center for the Evaluation of Risks to Human Reproduction (CERHR), concluding that there was minor concern for adverse reproductive effects in exposed men, but the data in women were insufficient [6]. BBP has been suggested to have an etiological association with endometriosis, as the severity of this condition and BBP concentration in blood have been strongly correlated [7]. Moreover, urinary concentrations of phthalate metabolites in pregnant mothers have been inversely correlated to anogenital distance among male infants, providing a demonstration of subtle developmental effects of prenatal exposure to phthalates in humans [8]. Fetal life is considered to be the most sensitive stage to the potential developmental and reproductive toxicity of the phthalates, but data on human fetal exposure to phthalates is still scarce [9].

All these lines of evidence point out BBP as an endocrine disrupting chemical, suggesting that it may interfere with the endocrine system and thus disrupt normal function in hormone-sensitive organs [10]. Several mechanisms have been proposed for the antiandrogenic effect of BBP in males, such as the decrease of testosterone by changes in the gene expression of genes related to the hormone synthesis and transport [11-13], or the down-regulation of Insl3 in the Leydig cells, which has a direct role in testis descent into the scrotal sac [14]. Although there is little data regarding the effects of BBP in females, other phthalates have been observed to decrease serum estradiol levels, prolong estrous cycles, and cause anovulation in adult cycling rats, acting through a receptor-mediated signaling pathway to suppress estradiol production in the ovary [15]. Endocrine disruptors may have an effect in other hormone-sensitive organs, such as the mammary gland. The breast tissue is strongly influenced by estrogens, which have an essential role in promoting the proliferation of both the normal and the neoplastic mammary epithelium. The response of the mammary gland to hormonal influences results in developmental changes that can permanently modify the architecture and the biological characteristics of the gland, and changes in the estrogenic environment during the critical stages of development can play a role in the future susceptibility to develop breast cancer [16,17]. Elevated estrogenic exposure *in utero *has been associated to an increase in breast cancer risk in both humans and animal models [10,18]. We have previously reported that neonatal and prepubertal exposure to BBP has little influence in the morphology of the mammary gland yet induced transitory changes in the gene expression profile of the gland [19], but new data is needed in relation to *in utero *exposure. More studies on mammary cancer susceptibility can provide an insight into the risk of breast cancer, the most common type of malignancy in women worldwide, whose rates are increasing in most countries [20]. The Sprague-Dawley rat is a good model for the study of breast cancer, and we have previously reported the validity of this model for the study of breast cancer in women [21]. The present work investigated the effect of prenatal exposure of BBP on the rat mammary gland. Fetuses were exposed to the BBP via oral gavage (120 mg or 500 mg BBP/body weight) given to their pregnant mothers, hence it is estimated that they receive 1/100 to 1/1000 of the mother dose. This approximation places this exposure near the EPA safe dose for humans of 0.2 mg/kg/day [22]. Then, we have used morphological parameters of gland differentiation [16,17], proliferative index, and genomic signature as biomarkers for detecting how prenatal exposure affects the post-natal development of the mammary gland.

## Methods

### Animals and experimental design

All animal studies were conducted in the University of Alabama at Birmingham in accordance with Institutional Guidelines for Animal Use and Care. Eight week old female Sprague Dawley CD rats (Charles River, Raleigh NC) were bred and maintained on phytoestrogen-free AIN-93G diet (Harlan Teklad, Madison WI) at the University of Alabama at Birmingham animal facility. For prenatal exposure, pregnant female rats (10/treatment group) were gastrically intubated with 120 mg BBP/kg body weight (BW), 500 mg BBP/BW, or an equivalent volume of sesame oil on days 10-21 post-conception. The BBP was obtained from Aldrich with 98% of purity, and it was dissolved in sesame oil. The doses administered were chosen based on studies by Singletary et al. [23] where they reported that treatment of female rats with 250 and 500 mg/kg of BBP for 7 days prior to dosing with DMBA decreased mammary tumor incidence. The LOAEL (lowest-observable-adverse-effect levels) of this compound has been determined at 250mg/kg/day [12]. The offspring were weaned at day 21. Body weight was recorded and vaginal opening was assessed as an index of maturation.

Female offspring were euthanized at 21, 35, 50 and 100 days of age. For the later three ages, all females were sacrificed in the estrus phase of the estrous cycle. The animals were anesthetized with ketamine/xylazine and the fourth mammary glands were promplty dissected. The "mammary tree" was trimmed free of excess fat and frozen for later microarrays analyses (1 female offspring from each litter at each time point). Transsection of the aorta was performed after the tissues were properly collected. From another set of randomly selected females from each litter, mammary glands were dissected for whole mount preparation and gland differentiation analysis, and the contralateral mammary glands were paraffin-blocked for determination of proliferative index. Ten mammary glands from different animals (from different litters) per group at each time point were used for each of the morphological, proliferation and molecular analyses.

### Morphological analysis of the mammary gland

The histoarchitecture of the mammary gland was studied from the whole-mounted tissue. The excised glands were fixed in 10% neutral-buffered formalin, defatted in acetone, re-hydrated and stained in alum carmine. After the staining, the glands were dehydrated in a series of graded alcohols, cleared in xylene and coverslipped with mounting media. The total number of epithelial structures (terminal end buds -TEB-, terminal ducts -TD-, alveolar buds -AB- and lobules type 1 -Lob1-) was determined under an Olympus microscope using a 40x magnification objective. These structures were counted in the zone C (5 mm-exterior zone), opposite to the nipple, which is the most actively growing area of the mammary gland [24].

### Study of the proliferative index

Animals were injected i.p. with bromodeoxyuridine (BrdU, Sigma, St. Louis, MO) two hours before euthanasia. Cycling animals received the BrdU injection while in estrus. The abdominal mammary glands were fixed in 10% neutral-buffered formalin, embedded in paraffin and sectioned at 4 μm thickness. Tissue sections were mounted on positively charged slides and immunochemically reacted with anti-BrdU monoclonal antibody (BioGenex, San Ramon, CA) using an automatic slide stainer (BioGenex). Incorporated BrdU was visualized using the streptavidin-biotin labeling system with 3, 3'-diaminobenzidine (DAB) as a color reaction substrate (BioGenex). The proliferative index was determined quantitatively under Olympus BX40 microscope (60x magnification objective) as the percentage of DAB positive cells within specific epithelial structures, i.e., TEB, TD and ducts, AB and Lob1. A minimum of 1,000 cells per structure in each sample was counted.

### Characterization of the gene expression profile

Total RNA from the mammary glands was extracted using the RNeasy Mini Kit (Qiagen Inc., Valencia, CA). The quantity and integrity of the samples were determined with NanoDrop 2.5.4. (NanoDrop Technologies, Wilmingon, DE) and by capillary electrophoresis using Agilent 2100 Bioanalyzer (Agilent Technologies, Palo Alto, CA). High quality RNA with absorbance 260/280 greater than 1.8 and RNA integrity number greater than 8 was used for further analysis. The ten RNA samples from each group at each time point were reduced to four different pooled samples for cDNA microarrays hybridization. Fluorescent cRNA probes were synthesized with the Agilent Low Input RNA Fluorescent Linear Amplification Kit (Agilent Technologies) in the presence of Cy3-dCTP or Cy5-dCTP (Perkin Elmer, Wellesley, MA), and purified using the RNeasy Mini Kit (Qiagen Inc., Valencia, CA). Agilent 60-mer oligo-microarrays containing 22,000 sequences were hybridized with 750 ng of Cy3 labeled control probe and 750 ng of Cy5 labeled BBP group probe at 60°C for 18 hours. Slides were washed, scanned, and submitted to Feature Extraction software v.9.0 (Agilent Technologies) using the defaults settings for background correction and normalization. The processed signals acquired from Feature Extraction were used for statistical analysis and expression values of individual arrays were combined by the average across the biological replicates. Spots with any quality issues were omitted from the analysis. To identify the genes differentially expressed each group treated with BBP was compared to its age-matched untreated control using empirical Bayes moderated t-test, implemented in the Bioconductor package "limma" [25,26]. For each comparison, all probes that presented at least 1.5-fold change (0.6 logarithm in base two) and with False Discovery Rate (FDR) less than 0.05 were considered statistically significant. The up- or down-regulated genes were classified by their biological processes according to Gene Ontology (GO) database and tested for over-representation using the Protein ANalysis THrough Evolutionary Relationships (PANTHER) functional analysis tool. Each list was statistically compared to NCBI: R. norvegicus genes reference list using the binomial test and the Bonferroni correction for multiple testing.

### Real time RT-PCR

To validate results of the microarrays we analyzed the expression of several genes found differentially expressed in the mammary glands of rats exposed prenatally to BBP by real-time reverse transcription PCR. All reactions were performed on the ABI Prism 7000 Sequence Detection System (Applied Biosystems, Foster City, CA) using the fluorescent TaqMan methodology (TaqMan One Step RT-PCR Master Mix Reagents, Applied Biosystems). The kit employs AmpliTaq Gold^® ^DNA polymerase for enhanced performance, Passive Reference I, and optimized buffer components. 100 ng of total RNA from individual samples was used for each reaction in a total volume of 50 μl according the manufacturer's protocol. The thermal cycling conditions comprised 30 min at 48°C, 10 min at 95°C, and 40 cycles of 15 s denaturation at 95°C and 60 s annealing at 60°C. Each gene was normalized using beta-actin as a control gene.

### Statistical methods

Statistical analyses were performed with the SPSS software (version 15.0). Since variables followed a normal distribution, determined by the Kolmogorov-Smirnov test, and had the equality of variances among groups, determined by Levene's test, parametric analyses were carried out. Results from body weight analysis, vaginal opening, mammary gland morphology and proliferation were analyzed using ANOVA followed by Tukey's post hoc test for multiple comparisons. Analysis for real time PCR was performed using two-sample two-tailed unpaired *t*-test. Data for microarrays was analyzed as indicated above. Differences were considered significant when P < 0.05.

## Results

### Effects of *in utero *exposure of BBP on body weights and sexual maturation

*In utero *exposure to low or high dose of BBP did not modify the body weight at 21, 35, 50 or 100 post-natal days (data not shown), or at day of vaginal opening. However, exposure to high dose of BBP significantly retarded the vaginal opening by two days (Table 1).

**Table 1:** Maturation parameters of female rats exposed prenatally to low (120 mg/kg BW) or high (500 mg/kg BW) dose of BBP

	Control	BBP low dose	BBP high dose
Day of vaginal opening	31.46 ± 0.55	31.30 ± 0.30	33.28 ± 0.29*

Body weight (g) at day of vaginal opening	116.8 ± 3.7	116.8 ± 4.0	119.1 ± 4.0

Values are mean ± standard error of the mean. * p < 0.05, significantly different from control.

### Effects of *in utero *exposure of BBP on mammary gland morphology

**Figure 1:**
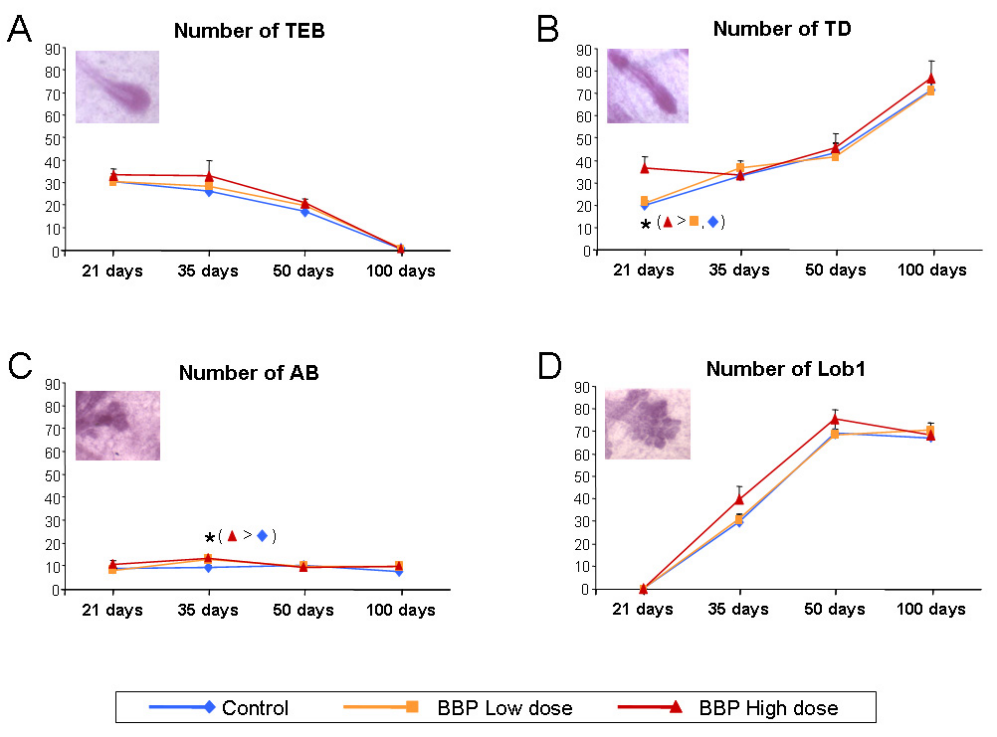
**Morphological analyses of the mammary gland**. Number of epithelial structures, i.e. TEB (A), TD (B), AB (C) and Lob1 (D), at different ages in the mammary gland of rats exposed prenatally to low or high dose of BBP. *: significant differences (p < 0.05).

The number of terminal ductal structures, i.e., TEB, AB, TD, and Lob 1, were analyzed in whole-mounted mammary glands at 21, 35, 50 and 100 post-natal days. The number of undifferentiated TEB decreased from 21 to 100 days in all groups (Figure 1A), while the TD increased over time (Figure 1B). The amount of AB began to increase at 21 days and reached a peak by 35 day decreasing thereafter (Figure 1C). The number of Lob1 also increased from 21 to 50 days, remaining at about the same number through 100 days (Figure 1D). Prenatal exposure to the high dose of BBP increased the number of TD in relation to control (p = 0.003) and the low dose group (p = 0.005) at 21 days, as well as it increased the number of AB in comparison with the control group (p = 0.02) at 35 days. The high dose group also showed a trend to increase the number of TEB by 50 days, but this result was not statistically significant (p = 0.093).

### Effects of *in utero *exposure of BBP on proliferative index

**Figure 2:**
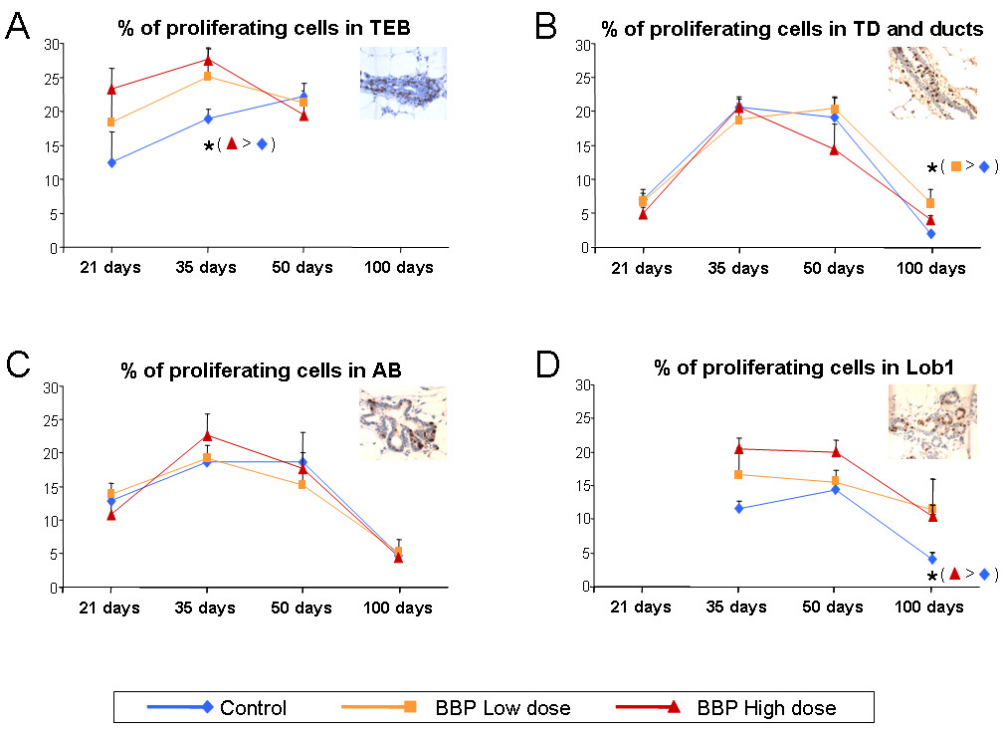
**Proliferative index in the mammary gland**. Percentage of proliferating cells in TEB (A), TD and ducts (B), AB (C) and Lob1 (D) at different ages in the mammary gland of rats exposed prenatally to low or high dose of BBP. Proliferating cells were identified by immunohistochemical detection of BrdU incorporation (brown cells). Olympus BX40 microscope with 40x objective. *: significant differences (p < 0.05).

The percentage of proliferating cells, as detected by the incorporation of BrdU into the newly synthesized DNA, is shown in Figure 2. By 21 days of age the TEB was the epithelial structure with the highest proliferation index in all groups. The proliferation of this structure was higher in the high dose group than in the control group by 21 (close to significance, p = 0.067) and 35 days (p = 0.010) (Figure 2A). The proliferative index of TEB by 100 days could not be determined since the presence of this structure at this age was too low to be detected (Figure 2A). The percentage of proliferating cells in TD and ducts was lower by 21 days, had increased by 35 and 50 days, and decreased by 100 days; at this last age the values from low dose group were higher compared to those in the control group (Figure 2B). There were not significant differences in the proliferative index in AB among the BBP-treated and control groups (Figure 2C). In Lob1 the percentage of proliferating cells were high by 35 and 50 days, and decreased in 100 days-old animals from all groups. At this last age the proliferative index was significantly higher in the BBP exposed group (high dose) than in controls (p = 0.004) (Figure 2D).

### Gene expression analysis by microarrays

The gene expression analysis of the mammary glands from prenatally BBP-treated rats showed differentially expressed genes by 35 days of age. Thus, at that age the low dose induced the up-regulation of 50 genes and decreased the expression of 335 genes, while the high dose of BBP increased the expression of 561 genes and down-regulated 799 genes. The known genes found as differentially expressed by effect of low and high dose are shown in Additional File 1, Table S1 and Additional File 2, Table S2, respectively. When analyses were performed at FDR < 0.1 we found 104 up-regulated and 169 down-regulated genes at PND21 in the mammary glands of the animals exposed to the low dose of BBP (data not shown).

The function of the genes differentially expressed was annotated according to Gene Ontology database. The main functional categories that were significantly (p < 0.05) over-represented for each cluster of genes when compared to the gene database for *Rattus norvegicus *are indicated in Table 2 (low dose of BBP) and Table 3 (high dose of BBP). Additional File 3, Table S3 details all functional categories found significantly over- or under-represented in the list of genes modulated by both exposures to BBP.

**Table 2:** Functional categories significantly over-represented in the up- or down-modulated genes at 35 days of age by the effects of *in utero *exposure to low dose of BBP

	Function	GO	p-value
Up-modulated	signal transduction	GO:0007165	6.86E-04
		GO:0007242	
	
	cell communication	GO:0007154	9.62E-04
	
	metabolic process	GO:0008152	2.64E-03
		GO:0044238	
	
	apoptosis	GO:0006915	3.68E-03
	
	transport	GO:0006810	6.42E-03
	
	cellular process	GO:0009987	7.55E-03
	
	other	GO:0008015	1.30E-03
		GO:0006936	1.34E-03

Down-modulated	primary metabolic process	GO:0044238	2.00E-06
		GO:0008152, GO:0006139, GO:0005975, GO:0019538	
	
	cellular process	GO:0009987	3.63E-05
	
	transport	GO:0006810	1.44E-04
		GO:0006886, GO:0015031, GO:0006897, GO:0016192	
	
	cell cycle	GO:0007049	1.84E-03
		GO:0007067	
	
	immune system process	GO:0002376	2.64E-03
	
	cell-matrix adhesion	GO:0007160	3.05E-03
	
	apoptosis	GO:0006915	4.63E-03
	
	other	GO:0007269	5.80E-03
		GO:0007059	6.14E-03

GO, Gene Ontology class.

**Table 3:** Functional categories significantly over-represented in the up- or down-modulated genes at 35 days of age by the effects of *in utero *exposure to high dose of BBP

	Function	GO	p-value
Up-modulated	metabolic process	GO:0008152,	6.71E-14
		GO:0044238, GO:0006629, GO:0005975, GO:0019538, GO:0006800, GO:0006807, GO:0006519, GO:0006732, GO:0006091, GO:0006766, GO:0006790, GO:0022904	
	
	cell communication	GO:0007154	1.04E-09
	
	signaling	GO:0007165	2.78E-09
		GO:0007242, GO:0007267, GO:0007166	
	
	transport	GO:0006810	5.14E-09
		GO:0006886, GO:0015031, GO:0006897, GO:0006869, GO:0016192, GO:0006811, GO:0051180, GO:0008643, GO:0006812	
	
	immune system process	GO:0002376	4.12E-08
	
	cellular process	GO:0009987	1.73E-07
	
	cell adhesion	GO:0007155	4.50E-07
		GO:0007160, GO:0016337	
	
	system process	GO:0003008	8.59E-07
		GO:0050877, GO:0008015, GO:0007601, GO:0007268	
	
	response to stimulus	GO:0050896	3.96E-05
		GO:0009605, GO:0006950, GO:0009636	
	
	homeostatic process	GO:0042592	1.22E-04
		GO:0007596,GO:0001678	
	
	developmental process	GO:0032502	5.03E-04
		GO:0009948, GO:0007399, GO:0048731, GO:0007398	
	
	apoptosis	GO:0006915	1.91E-03

Down-modulated	cellular process	GO:0009987	5.88E-18
	
	metabolic process	GO:0008152	1.14E-17
		GO:0044238, GO:0006139, GO:0019538, GO:0005975, GO:0006091, GO:0022904	
	
	cell cycle	GO:0007049	6.21E-13
		GO:0007067	
	
	cell communication	GO:0007154	2.02E-09
		GO:0007269	
	
	intracellular signaling cascade	GO:0007242	1.39E-08
		GO:0007165, GO:0007267, GO:0007166	
	
	cell motion	GO:0006928	8.25E-07
	
	transport	GO:0006810	5.83E-06
		GO:0008643, GO:0006886, GO:0015031, GO:0016192	
	
	cellular component organization	GO:0016043	2.56E-05
		GO:0006325, GO:0006996	
	
	apoptosis	GO:0006915	9.20E-05
		GO:0043066	
	
	immune system process	GO:0002376	1.07E-04
	
	developmental process	GO:0032502	5.31E-04
		GO:0048731, GO:0007399, GO:0007398	
	
	cell adhesion	GO:0007155	5.75E-04
		GO:0016337, GO:0007160	
	
	response to stimulus	GO:0050896	2.18E-03
		GO:0006950	
	
	system process	GO:0003008	2.45E-03
		GO:0050877, GO:0007601, GO:0007268	
	
	Other	GO:0000910	2.32E-03
		GO:0007276	7.53E-03

GO, Gene Ontology class.

### Gene expression analysis by real time RT-PCR

For validation of the sequences found differentially expressed in the analyses of the microarrays, we performed real time RT-PCR of a number of genes that are related to several functional categories significantly over-represented, including those involved in developmental process (specifically mammary gland differentiation, such as Csn3, Lalba, Mfge8, and Wap), proliferation (Bhlhb3), immune system (Cd24, Cd5, Ctse, Ptprc, and Slpi), lipid metabolism (Thedc1), signal transduction (Prkcq) and response to stress (Ddit4 and Cryab). Several of those genes were also annotated in other functional categories, such as cell adhesion (Mfge8, Cd5), apoptosis (Lalba, Prkcq, Ddit4, Cryab), transport (Mfge8, Cd5) or other metabolic processes (Slpi, Cryab).

**Figure 3:**
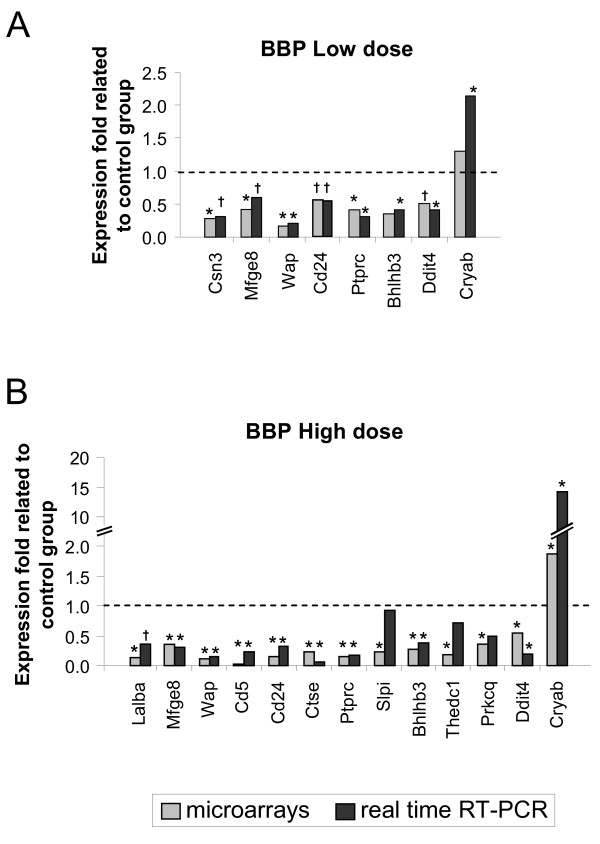
**Gene expression analyses of the mammary gland by real time PCR**. Gene expression analyses by real time RT-PCR and comparison with results from microarrays. Grey bars represent results obtained in the microarrays, black bars represent results obtained in the real time RT-PCR. Results show the mean fold expression of each treated group related to the mean of the control group. *: statistically significant changes (p < 0.05); †: changes close to significance (0.1 > p > 0.05).

Results of the real time RT-PCR analyses are shown in Figure 3. Overall, both doses decreased mammary differentiation markers (Csn3, Mfge8, Lalba or Wap), immune related genes, especially the high dose group (Cd24, Ptprc, Cd5 or Ctse), Bhlhb3 and Ddit4. Both doses also increased significantly the expression of Cryab.

## Discussion

In this study we found that *in utero *exposure to BBP significantly affects post-natal maturation of female rats, including delayed vaginal opening, changes in morphology of the mammary gland, and changes in gene expression of the mammary gland at puberty. These effects were dose-dependent, and the alterations of the mammary gland were age-specific.

Our findings demonstrate that prenatal exposure of BBP causes a significant delay in female sexual maturity as determined by the day of vaginal opening associated with a delayed pubertal onset. Other authors have reported changes in the time of vaginal opening by prenatal plus prepubertal exposure to BBP by administration to the mothers of 1g/L in drinking water [27], as well as modifications in the anogenital distance of rats exposed prenatally by oral gavage to the pregnant dams with 100 mg BBP/kg body weight [28]. Another indicator of sexual maturity is the morphological and cellular changes of mammary gland. We observed that rats exposed prenatally to the higher dose of BBP had a higher number of undifferentiated epithelial structures in the mammary gland mainly at the age of pubertal onset (35 days). This is in accordance with the delay in the time of vaginal opening and may suggest changes in the normal development of the mammary gland. Moreover, we found that in all epithelial structures, except in TD, the proliferative index in BBP-exposed animals was maximal by 35 days, in contrast to control animals, which had the highest proliferation indexes by 50 days. More importantly, exposure to the high dose of BBP induced more proliferating TEB by 35 days when compared to unexposed animals. The TEB is the least differentiated epithelial structure in the mammary gland, and the most susceptible to malignant transformation [29]. Thus, alterations in number and/or proliferation of TEB may cause the changes in susceptibility of this gland to malignant transformation. For instance, we have previously demonstrated that both pregnancy and treatment of virgin rats with the placental hormone human chorionic gonadotropin (hCG) protected rat mammary gland from chemically-induced malignant transformation and that this effect was resulted from elimination of undifferentiated TEB and inhibition of cell proliferation [16,17]. It has been described that the age of highest susceptibility of this gland to chemically-induced carcinogenesis is around 50 days, when the TEB are more proliferative [17]. The fact that the BBP-exposed animals showed more TEB and AB and more proliferating cells by 35 days suggests that BBP can induce alterations in the development of the mammary tissues, expanding or shifting the window of susceptibility of the gland to transformation.

The changes in the susceptibility of the mammary gland to chemically-induced carcinogenesis are likely resulted from the alterations in specific genomic signature. Thus, we have studied the gene expression profiles of the mammary glands from the rats exposed prenatally to the low and the high doses of BBP. In accordance to the results of puberty onset, morphology and proliferative index of the mammary gland, we found changes in the gene expression profile of the glands by 35 days of age. The effect of xenobiotics with a putative endocrine-disrupting influence seems to depend on time of exposure. Thus, we previously observed that treatment with the same high dose of BBP during the neonatal and lactational periods caused transitory changes in the gene expression profile of the gland, inducing a high number of up-modulated genes by 21 days of age (just after the end of the exposure) but not at other ages, and no changes in the day of vaginal opening [19]. *In utero *exposure to 250 μg of bisphenol A (BPA), another endocrine disruptor widely used, also induced time-specific changes in the gene expression profile of the mammary gland [30], protein expression [31], and shifted the windows of susceptibility for mammary carcinogenesis [32]. Different effects were observed when the same dose was administrated during the lactational period (neonatal + prepubertal), finding increased proliferation and decreased apoptosis in the mammary glands at 50 days, but not at 21 days [33].

The function of genes dysregulated at puberty by effect of *in utero *exposure to BBP included developmental and immune system processes, metabolism, signaling or apoptosis. For example, we observed a down-modulation of the immune-related genes Cd5, Cd3d, Ptprc, especially by effect of the higher dose. Other authors have also suggested an influence of phthalates on immunity. BBP inhibited lipopolysaccharide-induced TNF-alpha in a mouse macrophage cell line [34], and human case-control data have associated the concentration of BBP in house dust with allergic symptoms [35]. We also observed at 35 days a down-modulation of genes related to developmental processes in the BBP-exposed rats, including mammary gland differentiation markers (casein kappa -Csn3-, lactalbumin alpha -Lalba-, whey acidic protein -Wap- and milk fat globule-EGF factor 8 protein -Mfge8 or lactadherin-). Other genes annotated to "cell proliferation and differentiation" category have been found down-regulated at that age. That is the case of Bhlhb3, a transcription factor that is involved in circadian rhythm, differentiation, and that has been reported to repress the transcription of cyclin D1 and thus inhibit proliferation [36]. These results are in agreement with the ones obtained in the proliferation analysis (more proliferative structures by 35 days in the BBP-exposed groups). In relation to this, we have also found a down-modulation of genes related to apoptosis (such as lactalbumin [37]) and up-modulation of Cryab, which has been described to have anti-apoptotic effects [38]. Hence, our results are compatible with a decreased apoptosis, as it has been described for other endocrine disruptors such as BPA [33]. All these data suggest a proliferation/apoptosis balance tilted to proliferation in the mammary glands of the exposed rats, and that TEB may be more susceptible to malignant transformation at 35 days of age.

Prenatal BBP treatment also induced modifications in the expression of genes related to response to stress (Ddit4, Cryab) and metabolism. The down-modulation of Thedc1, with a role in lipid metabolism, was not confirmed by real time PCR at 35 days although we found a significant increase in its expression at 50 days (data not shown). Moreover, some of the genes studied, such as Cryab, had also a role in metabolism [39]. Modifications in the expression of similar genes (related to metabolism or oxidative stress) have been also described in testis of rats exposed prenatally with BBP. Such modifications are suggested to be related with changes in the hormonal balance of developing fetal testis [11]. Considering such evidence, our results could also be a reflection, at least in part, of changes in the hormonal balance in the BBP treated rats.

Therefore, our data suggest that prenatal exposure to BBP can have an effect on normal post-natal development of the rat. Delayed vaginal opening could be the result of an altered maturation of the hypothalamic-gonadal axis for the timing of the onset of puberty. While the development of the mammary gland undergoes a critical phase with the initiation of puberty, modifications in the morphology of the gland and in the quantity of each specific structure begins before puberty and continues for a long period of time. The changes in the morphology and the proliferative index of the mammary gland, more than advanced or retarded growth, suggest a different pattern in the normal development of the gland that would affect the windows of susceptibility of this gland. This would increase or decrease the susceptibility to malignant transformation depending of the moment when a carcinogenic insult occurs. Such changes can be supported, at least in part, by modifications in the gene expression profile of the gland. BBP exposure down-modulated the expression of genes related to functions like immunity, apoptosis and also differentiation markers, which would be in accordance with a less differentiated gland. Our results also points out the possibility of a metabolic disruption of this compound. In fact, there is growing evidence that several pollutants, some of them considered endocrine disruptors such as phthalates, may interact with members of the PPAR superfamily and interfere with lipid and carbohydrate metabolism [40]. Such pollutants are tentatively named "metabolic disrupters", and are currently of interest since several human diseases, such as atherosclerosis, hyperlipidemia and obesity, often reflect subtle deregulations of complex metabolic pathways and are manifested after many years [41].

On the other hand, we need to consider the different methodological approaches in the interpretation of the data obtained. We have visualized the number of the epithelial structures and the proliferation index of such structures. However, because stromal-epithelial interactions are essential for the development of the mammary gland, in addition to methodological reasons, gene expression profile was determined in the whole mammary tissue of the forth gland (with the exception of the lymph node). Thus, the changes in gene expression observed can be related to modifications in the biology of the epithelial (as suggested by the mammary differentiation markers), and stromal cells, such as the fibroblasts or adipocytes. Actually, changes in lipid metabolism may be occurring in the adipose tissue, and changes in the immune related genes may be a reflection of modifications in the number, type or function of infiltrated immune cells. In any case, all these cell types have an essential role in the development of the mammary gland, and thus changes in their expression profile may be of importance in the biology of such tissue [42,43].

As mentioned above, we have previously reported that the prenatal exposure to another endocrine disruptor, bisphenol A (BPA), also induced changes in the mammary gland that were time- and dose-specific [30]. Both doses tested (25 and 250 μg/kg) caused modification of the expression of similar clusters of genes (e.g. several differentiation markers, immune-related genes) that have also been reported as differentially modulated by BBP exposure in this work. These evidences suggest that some genes involved in general and essential processes may be especially sensitive to the effect of endocrine disruptors, and taken together, data regarding mammary gland morphology, proliferative index and gene expression profile analyses suggest changes in the biology of the gland that may change its susceptibility to breast cancer. Even when caution must be applied when comparing animal studies with human data, these studies give further evidence of the potential effect of BBP on human health. Although the action of this and other xenobiotics may not be as strong as other steroidal endocrine disruptors, they are produced in much larger quantities and population is continuously exposed to them. Actually BBP exposure in adults has been estimated at 2 μg/kg body weight/day, and three-old higher in infants and children [44]. Moreover, urinary concentrations of BBP have been detected in > 75% of reference population, with significantly higher levels in children and adolescents [45]. The mechanisms by which this xenobiotic alters mammary gland biology and gene expression are not still well determined. New studies are currently underway to elucidate if there are modifications in the hormonal balance causing such changes and those are related to the risk of mammary gland malignant transformation.

## Conclusions

The results of this work suggest that prenatal exposure to BBP can have an effect on normal post-natal development of the rat, as characterized by delayed vaginal opening and modifications in morphology, proliferation index and gene expression in the mammary gland. Morphology and proliferative rate of this gland suggested changes in its pattern of development that could affect the windows of susceptibility to transformation. Prenatal BBP exposure also induced changes in the gene expression of the mammary gland long after the end of the exposure, mainly at the beginning of puberty. Genes related to immune function, cell signaling, proliferation and differentiation seem to be particularly sensitive to the effect of BBP. All these data may be, on the one hand, a reflection of endocrine disruption, and on the other, an indicator of changes in the risk of breast cancer.

## List of abbreviations

AB: alveolar buds; BBP: butyl benzyl phthalate; BPA: bisphenol A; BrdU: bromodeoxyuridine; BW: body weight; DAB: diaminobenzidine; FDR: false discovery rate; GO: gene ontology; Lob1: lobule type 1; RT-PCR: reverse transcription polymerase chain reaction; TD: terminal ducts; TEB: terminal end bud.

## Competing interests

The authors declare that they have no competing interests.

## Authors' contributions

RM performed the molecular experiments, data analyses and contributed in the writing of the manuscript. JSP participated in the molecular experiments, gene expression analyses and the writing of the article. RW carried out the morphological and proliferative index analyses. IHR participated in the design and coordination of the study, and helped in the writing of the manuscript. CAL participated in the coordination of the study and performed the animal assays. JR conceived of the study, coordinated the work and participated in the writing of the manuscript. All authors read and approved the final manuscript.

## Supplementary Material

Additional file 1 **Modulated genes by effect of *in utero *exposure to low dose of BBP**. List of known up- and down-regulated genes at 35 days of age in mammary glands of rats exposed prenatally to low dose (120 mg/kg BW) of BBP. For each gene the name, symbol, GeneBank accession number, fold change expression value (in Log_2_) versus control group, and false discovery rate (FDR) is indicated. Click here for file

Additional file 2 **Modulated genes by effect of *in utero *exposure to high dose of BBP**. List of known up- and down-regulated genes at 35 days of age in mammary glands of rats exposed prenatally to high dose (500 mg/kg BW) of BBP. For each gene the name, symbol, GeneBank accession number, fold change expression value (in Log_2_) versus control group, and false discovery rate (FDR) is indicated. Click here for file

Additional file 3 **Functional categories analysis of the modulated genes by effect of *in utero *exposure to BBP**. Detailed functional categories significantly over- or under-represented in the list of genes found as modulated at 35 days of age by the effects of *in utero *exposure to low (120 mg/kg BW) and high dose high dose (500 mg/kg BW) of BBP. Click here for file
