# Supplementary material for: In utero exposure to butyl benzyl phthalate induces modifications in the morphology and the gene expression profile of the mammary gland: an experimental study in rats
Source: Environ Health. 2011 Jan 17;10:5. doi: 10.1186/1476-069X-10-5 (PMC3033239; doi:10.1186/1476-069X-10-5)
Supplement: Additional file 1 — Modulated genes by effect of in utero exposure to low dose of BBP. List of known up- and down-regulated genes at 35 days of age in mammary glands of rats exposed prenatally to low dose (120 mg/kg BW) of BBP. For each gene the name, symbol, GeneBank accession number, fold change expression value (in Log2) versus control group, and false discovery rate (FDR) is indicated. [file 1476-069X-10-5-S1.DOC]

**Additional Table 1.**

List of known up- and down-regulated genes at 35 days of age in mammary glands of rats exposed prenatally to low dose (120 mg/kg BW) of BBP. For each gene the name, symbol, GeneBank accession number, fold change expression value (in Log_2_) versus control group, and false discovery rate (FDR) is indicated.

| **Gene Name** | **Symbol** | **Accesion Nº** | ***Log_2_*** | **FDR** |
| --- | --- | --- | --- | --- |
| **Up-modulated:** |  |  |  |  |
| ADAM metallopeptidase domain 32 | AJ131563 | Adam32 | 0.768 | 0.038 |
| adrenergic, alpha-1B-, receptor | NM_016991 | Adra1b | 0.840 | 0.035 |
| aldehyde dehydrogenase 6 family, member A1 | NM_031057 | Aldh6a1 | 0.864 | 0.048 |
| annexin A1 | NM_012904 | Anxa1 | 0.857 | 0.037 |
| antigen p97 (melanoma associated) identified by monoclonal antibodies 133.2 and 96.5 | XM_237839 | Mfi2 | 0.694 | 0.048 |
| aquaporin 7 | NM_019157 | Aqp7 | 0.746 | 0.047 |
| aspartoacylase | NM_024399 | Aspa | 1.363 | 0.039 |
| carboxylesterase 2-like | NM_133586 | Ces2l | 0.674 | 0.048 |
| cell death-inducing DFFA-like effector a | XM_214551 | Cidea | 1.122 | 0.035 |
| conserved helix-loop-helix ubiquitous kinase | CB548045 | Chuk | 0.727 | 0.042 |
| core 1 synthase, glycoprotein-N-acetylgalactosamine 3-beta-galactosyltransferase, 1 | NM_022950 | C1galt1 | 0.745 | 0.037 |
| cytoplasmic polyadenylation element binding protein 1 | XM_218858 | Cpeb1 | 1.059 | 0.037 |
| epithelial membrane protein 1 | NM_012843 | Emp1 | 0.636 | 0.044 |
| fibroblast growth factor receptor-like 1 | NM_199114 | Fgfrl1 | 0.753 | 0.048 |
| G protein-coupled receptor 120 | XM_215281 | Gpr120 | 1.179 | 0.043 |
| holocarboxylase synthetase (biotin-(proprionyl-Coenzyme A-carboxylase (ATP-hydrolysing)) ligase) | XM_221630 | Hlcs | 0.628 | 0.045 |
| insulin-like growth factor binding protein 3 | NM_012588 | Igfbp3 | 0.800 | 0.035 |
| leucine rich repeat containing 8 family, member D | NM_001008338 | Lrrc8d | 1.139 | 0.038 |
| myogenin | NM_017115 | Myog | 0.922 | 0.043 |
| odz, odd Oz/ten-m homolog 4 (Drosophila) | XM_218927 | Odz4 | 1.086 | 0.037 |
| paraoxonase 3 | NM_001004086 | Pon3 | 0.976 | 0.035 |
| potassium voltage gated channel, Shab-related subfamily, member 1 | NM_013186 | Kcnb1 | 0.956 | 0.039 |
| potential ligand-binding protein | X60660 | RY2G5 | 0.822 | 0.039 |
| proprotein convertase subtilisin/kexin type 5 | L14933 | Pcsk5 | 0.799 | 0.044 |
| solute carrier family 46, member 2 | CB547809 | Slc46a2 | 0.614 | 0.048 |
| TDP-glucose 4,6-dehydratase | XM_224518 | Tgds | 0.772 | 0.035 |
|  |  |  |  |  |
| **Down-modulated:** |  |  |  |  |
| 1-acylglycerol-3-phosphate O-acyltransferase 4 (lysophosphatidic acid acyltransferase, delta) | NM_133406 | Agpat4 | -0.948 | 0.035 |
| 5'-3' exoribonuclease 1 | XM_217233 | Xrn1 | -1.074 | 0.036 |
| adipose differentiation related protein | NM_001007144 | Adfp | -0.746 | 0.039 |
| AFG3(ATPase family gene 3)-like 2 (yeast) | XM_225866 | Afg3l2 | -0.768 | 0.035 |
| aldehyde dehydrogenase 3 family, member B2 | CB545830 | Aldh3b2 | -1.028 | 0.037 |
| amyloid beta (A4) precursor protein-binding, family B, member 3 | NM_053957 | Apbb3 | -0.620 | 0.046 |
| ankyrin repeat and sterile alpha motif domain containing 3 | NM_001009676 | Anks3 | -0.810 | 0.037 |
| arylsulfatase A | XM_235566 | Arsa | -0.680 | 0.044 |
| ATP synthase, H+ transporting, mitochondrial F1 complex, gamma polypeptide 1 | CB545338 | Atp5c1 | -1.313 | 0.038 |
| bone marrow stromal cell antigen 2 | NM_198134 | Bst2 | -0.854 | 0.035 |
| brain-specific angiogenesis inhibitor 3 | XM_217367 | Bai3 | -0.695 | 0.037 |
| brix domain containing 1 | XM_215404 | Bxdc1 | -0.858 | 0.035 |
| bromodomain and WD repeat domain containing 2 | XM_219377 | Brwd2 | -1.657 | 0.035 |
| bromodomain containing 2 | NM_212495 | Brd2 | -0.981 | 0.035 |
| BTAF1 RNA polymerase II, B-TFIID transcription factor-associated, (Mot1 homolog, S. cerevisiae) | XM_347206 | Btaf1 | -0.626 | 0.046 |
| calpain 1 | U53858 | Capn1 | -0.944 | 0.048 |
| calpain 6 | NM_031808 | Capn6 | -1.265 | 0.047 |
| carbohydrate (keratan sulfate Gal-6) sulfotransferase 1 | NM_001011955 | Chst1 | -1.210 | 0.035 |
| casein kappa | NM_031562 | Csn3 | -1.827 | 0.050 |
| cathepsin Z | NM_183330 | Ctsz | -1.014 | 0.035 |
| CDC5 cell division cycle 5-like (S. pombe) | NM_053527 | Cdc5l | -0.975 | 0.035 |
| CDK5 regulatory subunit associated protein 1-like 1 | XM_341524 | Cdkal1 | -1.208 | 0.035 |
| CDP-diacylglycerol synthase 1 | NM_031242 | Cds1 | -0.948 | 0.035 |
| CDP-diacylglycerol--inositol 3-phosphatidyltransferase (phosphatidylinositol synthase) | NM_138899 | Cdipt | -0.818 | 0.035 |
| centaurin, delta 2 | BC088328 | Centd2 | -0.719 | 0.044 |
| CGRP receptor component | NM_053670 | Crcp | -0.752 | 0.048 |
| chemokine (C-X-C motif) ligand 16 | NM_001017478 | Cxcl16 | -0.753 | 0.039 |
| claudin 3 | NM_031700 | Cldn3 | -0.926 | 0.035 |
| cleavage stimulation factor, 3' pre-RNA subunit 2, tau | AW917690 | Cstf2t | -0.980 | 0.035 |
| Cnksr family member 3 | NM_001012061 | Cnksr3 | -0.983 | 0.035 |
| coiled-coil domain containing 130 | XM_222461 | Ccdc130 | -1.016 | 0.037 |
| copine VI | XM_240182 | Cpne6 | -0.847 | 0.037 |
| corepressor interacting with RBPJ, 1 | NM_001007799 | Cir1 | -0.714 | 0.046 |
| CTP synthase | XM_233467 | Ctps | -0.668 | 0.040 |
| cut-like homeobox 1 | XM_347163 | Cux1 | -1.170 | 0.047 |
| dachsous 1 (Drosophila) | XM_219128 | Dchs1 | -0.905 | 0.042 |
| DENN/MADD domain containing 5A | XM_219270 | Dennd5a | -0.967 | 0.035 |
| diablo homolog (Drosophila) | NM_001008292 | Diablo | -0.726 | 0.038 |
| dihydrolipoamide branched chain transacylase E2 | XM_342328 | Dbt | -0.939 | 0.049 |
| DnaJ (Hsp40) homolog, subfamily C, member 18 | NM_001013887 | Dnajc18 | -0.842 | 0.035 |
| dolichyl-phosphate mannosyltransferase polypeptide 2, regulatory subunit | NM_019252 | Dpm2 | -0.771 | 0.048 |
| E1A binding protein p300 | CB547347 | Ep300 | -0.942 | 0.042 |
| E2F transcription factor 5 | XM_574892 | E2f5 | -0.818 | 0.035 |
| EF-hand domain (C-terminal) containing 1 | BF564803 | Efhc1 | -1.121 | 0.035 |
| elastin microfibril interfacer 1 | XM_238447 | Emilin1 | -0.634 | 0.043 |
| enhancer of zeste homolog 2 (Drosophila) | XM_342680 | Ezh2 | -0.829 | 0.037 |
| epsin 3 | NM_001024791 | Epn3 | -1.075 | 0.035 |
| eukaryotic translation initiation factor 1A domain containing | NM_001008305 | Eif1ad | -0.802 | 0.035 |
| eukaryotic translation initiation factor 2B, subunit 2 beta | NM_032058 | Eif2b2 | -0.765 | 0.035 |
| exportin 7 | XM_341353 | Xpo7 | -1.185 | 0.035 |
| F-box and leucine-rich repeat protein 19 | XM_219356 | Fbxl19 | -0.903 | 0.037 |
| fibulin 2 | XM_232197 | Fbln2 | -0.811 | 0.048 |
| forkhead box M1 | NM_031633 | Foxm1 | -0.733 | 0.037 |
| Friend leukemia virus integration 1 | NM_001017381 | Fli1 | -0.751 | 0.043 |
| G protein-coupled receptor 160 | NM_001025147 | Gpr160 | -0.985 | 0.039 |
| G protein-coupled receptor 20 | NM_022216 | Gpr20 | -1.024 | 0.035 |
| gastrulation brain homeobox 1 | AF219999 | Gbx1 | -1.112 | 0.038 |
| GIY-YIG domain containing 2 | NM_001009292 | Giyd2 | -0.733 | 0.050 |
| glucosamine (UDP-N-acetyl)-2-epimerase/N-acetylmannosamine kinase | NM_053765 | Gne | -1.134 | 0.035 |
| glucosidase, alpha; neutral AB | XM_215144 | Ganab | -0.925 | 0.042 |
| glucuronidase, beta | NM_017015 | Gusb | -0.795 | 0.048 |
| glutamate decarboxylase 1 | NM_017007 | Gad1 | -3.118 | 0.035 |
| glutamate receptor, ionotropic, delta 2 (Grid2) interacting protein | XM_239604 | Grid2ip | -1.359 | 0.048 |
| glutamate receptor, ionotropic, N-methyl-D-aspartate 3B | NM_133308 | Grin3b | -1.057 | 0.037 |
| G-rich RNA sequence binding factor 1 | XM_223327 | Grsf1 | -0.715 | 0.043 |
| growth arrest specific 5 | U77829 | Gas5 | -0.730 | 0.048 |
| growth arrest specific 6 | BC070881 | Gas6 | -0.659 | 0.043 |
| H2A histone family, member X | XM_576399 | H2afx | -1.158 | 0.035 |
| HCCA2 protein | XM_574587 | Hcca2 | -0.780 | 0.037 |
| helicase with zinc finger domain | XM_237808 | Helz | -1.089 | 0.035 |
| hepsin | NM_017112 | Hpn | -1.626 | 0.035 |
| histone cluster 1, H4b | NM_022686 | Hist1h4b | -0.806 | 0.035 |
| HLA-B associated transcript 1 | NM_133300 | Bat1 | -0.768 | 0.046 |
| homeodomain interacting protein kinase 4 | NM_001024776 | Hipk4 | -2.172 | 0.035 |
| homer homolog 1 (Drosophila) | NM_031707 | Homer1 | -0.966 | 0.035 |
| hydroxysteroid (17-beta) dehydrogenase 11 | NM_001004209 | Hsd17b11 | -0.774 | 0.042 |
| hydroxysteroid (17-beta) dehydrogenase 8 | NM_212529 | Hsd17b8 | -0.605 | 0.048 |
| integrator complex subunit 10 | XM_214334 | Ints10 | -0.810 | 0.044 |
| integrin, beta 6 | NM_001004263 | Itgb6 | -1.033 | 0.047 |
| interferon kappa | BF561729 | Ifnk | -0.648 | 0.048 |
| interferon regulatory factor 6 | XM_344194 | Irf6 | -0.992 | 0.035 |
| intraflagellar transport 81 homolog (Chlamydomonas) | NM_199120 | Ift81 | -0.903 | 0.037 |
| isoleucyl-tRNA synthetase | XM_225196 | Iars | -0.806 | 0.048 |
| jagged 2 | XM_343119 | Jag2 | -0.961 | 0.038 |
| karyopherin alpha 2 | NM_053483 | Kpna2 | -0.611 | 0.042 |
| katanin p80 (WD repeat containing) subunit B 1 | XM_214635 | Katnb1 | -0.893 | 0.035 |
| kelch-like 5 (Drosophila) | XM_223418 | Klhl5 | -1.163 | 0.035 |
| kinesin family member 21A | XM_217022 | Kif21a | -1.036 | 0.035 |
| kinesin family member 2C | NM_134472 | Kif2c | -0.954 | 0.035 |
| KRAB-A domain containing 1 | XM_342681 | Krba1 | -1.082 | 0.043 |
| leucine-rich PPR-motif containing | NM_001008519 | Lrpprc | -0.628 | 0.040 |
| leukocyte receptor cluster (LRC) member 1 | XM_214797 | Leng1 | -1.496 | 0.035 |
| lin-37 homolog (C. elegans) | XM_214898 | Lin37 | -1.052 | 0.038 |
| lipase maturation factor 1 | XM_340769 | Lmf1 | -1.268 | 0.035 |
| low density lipoprotein-related protein 1 (alpha-2-macroglobulin receptor) | XM_243524 | Lrp1 | -0.832 | 0.047 |
| lysozyme-like 4 | XM_343507 | Lyzl4 | -0.790 | 0.038 |
| major facilitator superfamily domain containing 6 | BG665267 | Mfsd6 | -0.800 | 0.046 |
| Mdm4 p53 binding protein homolog (mouse) | NM_001012026 | Mdm4 | -0.796 | 0.042 |
| mediator complex subunit 13 | XM_220813 | Med13 | -0.698 | 0.037 |
| methyl CpG binding protein 2 | NM_022673 | Mecp2 | -0.947 | 0.038 |
| microtubule-associated protein 2 | U30938 | Map2 | -0.932 | 0.038 |
| milk fat globule-EGF factor 8 protein | NM_012811 | Mfge8 | -1.211 | 0.035 |
| mitochondrial ribosomal protein L9 | AW142434 | mrpl9 | -1.137 | 0.035 |
| mitochondrial ribosomal protein S15 | NM_001007653 | Mrps15 | -0.678 | 0.038 |
| mitochondrial ribosomal protein S5 | XM_215833 | Mrps5 | -0.751 | 0.044 |
| mitogen-activated protein kinase 8 interacting protein 3 | XM_220232 | Mapk8ip3 | -1.025 | 0.040 |
| mitogen-activated protein kinase kinase 1 interacting protein 1 | NM_001008375 | Map2k1ip1 | -0.863 | 0.037 |
| mitogen-activated protein kinase kinase kinase 11 | NM_001013150 | Map3k11 | -0.846 | 0.035 |
| M-phase phosphoprotein 10 (U3 small nucleolar ribonucleoprotein) | XM_238166 | Mphosph10 | -0.939 | 0.036 |
| mucin 1, cell surface associated | XM_342281 | Muc1 | -1.706 | 0.035 |
| mucolipin 1 | XM_213684 | Mcoln1 | -0.821 | 0.038 |
| multimerin 1 | CB547242 | Mmrn1 | -1.374 | 0.037 |
| MYST histone acetyltransferase 2 | NM_181081 | Myst2 | -0.763 | 0.037 |
| N-6 adenine-specific DNA methyltransferase 2 (putative) | XM_214202 | N6amt2 | -0.915 | 0.037 |
| NADPH oxidase 4 | NM_053524 | Nox4 | -0.636 | 0.048 |
| NCK adaptor protein 2 | XM_237115 | Nck2 | -0.625 | 0.049 |
| neuron navigator 2 | XM_341864 | Nav2 | -0.932 | 0.035 |
| neurotrophin receptor associated death domain | NM_139259 | Nradd | -0.955 | 0.038 |
| NLR family, CARD domain containing 4 | XM_216640 | Nlrc4 | -0.875 | 0.037 |
| NOL1/NOP2/Sun domain family, member 6 | XM_225588 | Nsun6 | -0.840 | 0.048 |
| nth (endonuclease III)-like 1 (E.coli) | XM_213228 | Nthl1 | -1.043 | 0.035 |
| nuclear factor of kappa light polypeptide gene enhancer in B-cells inhibitor, beta | NM_030867 | Nfkbib | -0.642 | 0.038 |
| nuclear transcription factor, X-box binding 1 | NM_001024784 | Nfx1 | -1.224 | 0.038 |
| nucleolar complex associated 3 homolog (S. cerevisiae) | XM_574664 | Noc3l | -0.888 | 0.039 |
| nucleoporin 107 | NM_053830 | Nup107 | -1.003 | 0.035 |
| NUF2, NDC80 kinetochore complex component, homolog (S. cerevisiae) | NM_001012028 | Nuf2 | -0.607 | 0.044 |
| oxidative stress induced growth inhibitor 1 | NM_138504 | Osgin1 | -1.006 | 0.039 |
| palmitoyl-protein thioesterase 1 | NM_022502 | Ppt1 | -0.687 | 0.042 |
| PAP associated domain containing 5 | XM_226334 | Papd5 | -0.736 | 0.048 |
| par-3 (partitioning defective 3) homolog (C. elegans) | NM_031235 | Pard3 | -0.992 | 0.035 |
| pepsinogen 5, group I | NM_021753 | Pga5 | -0.997 | 0.046 |
| peptidylprolyl isomerase F (cyclophilin F) | NM_172243 | Ppif | -0.731 | 0.048 |
| phosphatidylinositol 4-kinase type 2 beta | NM_001005883 | Pi4k2b | -0.865 | 0.035 |
| phosphatidylinositol-4-phosphate 5-kinase, type I, beta | NM_001012743 | Pip5k1b | -2.445 | 0.010 |
| phospholipid scramblase 1 | NM_057194 | Plscr1 | -0.850 | 0.038 |
| phosphoribosyl pyrophosphate synthetase-associated protein 1 | NM_022545 | Prpsap1 | -1.077 | 0.035 |
| pituitary tumor-transforming 1 | NM_022391 | Pttg1 | -1.080 | 0.035 |
| platelet derived growth factor C | NM_031317 | Pdgfc | -1.035 | 0.042 |
| polycomb group ring finger 1 | NM_001007000 | Pcgf1 | -1.277 | 0.035 |
| polymerase (DNA-directed), delta 3, accessory subunit | NM_001024750 | Pold3 | -0.773 | 0.037 |
| polynucleotide kinase 3'-phosphatase | NM_001004259 | Pnkp | -0.772 | 0.038 |
| potassium voltage-gated channel, shaker-related subfamily, beta member 1 | NM_017303 | Kcnab1 | -0.840 | 0.048 |
| processing of precursor 5, ribonuclease P/MRP subunit (S. cerevisiae) | XM_213794 | Pop5 | -1.194 | 0.045 |
| proteasome (prosome, macropain) subunit, beta type 2 | NM_017284 | Psmb2 | -0.909 | 0.035 |
| proteasome maturation protein | XM_213700 | Pomp | -1.069 | 0.035 |
| protein regulator of cytokinesis 1 | XM_218820 | Prc1 | -1.328 | 0.035 |
| R3H domain and coiled-coil containing 1 | XM_341347 | R3hcc1 | -1.191 | 0.035 |
| RAB25, member RAS oncogene family | XM_227404 | Rab25 | -0.958 | 0.035 |
| Ral GEF with PH domain and SH3 binding motif 2 | XM_222773 | Ralgps2 | -1.345 | 0.040 |
| RALBP1 associated Eps domain containing 1 | XM_214954 | Reps1 | -0.982 | 0.035 |
| RanBP-type and C3HC4-type zinc finger containing 1 | NM_021764 | Rbck1 | -1.401 | 0.035 |
| RAP1 interacting factor homolog (yeast) | BF287849 | Rif1 | -0.758 | 0.046 |
| ras homolog gene family, member G (rho G) | XM_218977 | Rhog | -0.981 | 0.047 |
| regulator of G-protein signaling 10 | XM_341936 | Rgs10 | -1.073 | 0.035 |
| regulatory factor X, 7 | XM_236392 | Rfx7 | -0.770 | 0.046 |
| renin binding protein | NM_031095 | Renbp | -1.425 | 0.035 |
| reticulocalbin 1, EF-hand calcium binding domain | XM_342481 | Rcn1 | -1.122 | 0.035 |
| retinoic acid receptor responder (tazarotene induced) 1 | NM_001014790 | Rarres1 | -0.787 | 0.045 |
| Rho GTPase activating protein 11A | XM_230459 | Arhgap11a | -0.857 | 0.035 |
| ribosomal protein L30 | NM_022699 | Rpl30 | -1.040 | 0.038 |
| ribosomal protein S21 | NM_031111 | Rps21 | -0.797 | 0.035 |
| ring finger and CCCH-type zinc finger domains 1 | XM_222801 | Rc3h1 | -0.854 | 0.037 |
| ring finger protein 32 | NM_001012095 | Rnf32 | -1.452 | 0.047 |
| ring finger protein 5 | CA339010 | Rnf5 | -1.055 | 0.047 |
| RNA binding motif protein, X-linked 2 | XM_346355 | Rbmx2 | -0.805 | 0.035 |
| SAP domain containing ribonucleoprotein | XM_343147 | Sarnp | -0.971 | 0.037 |
| secreted and transmembrane 1A | NM_001013043 | Sectm1a | -1.552 | 0.035 |
| sel-1 suppressor of lin-12-like (C. elegans) | BF546967 | Sel1l | -1.050 | 0.035 |
| serglycin | NM_020074 | Srgn | -0.923 | 0.035 |
| serine (or cysteine) peptidase inhibitor, clade E, member 1 | NM_012620 | Serpine1 | -0.666 | 0.048 |
| SH3 domain containing, Ysc84-like 1 (S. cerevisiae) | XM_343045 | Sh3yl1 | -0.722 | 0.050 |
| Sh3kbp1 binding protein 1 | XM_214873 | Shkbp1 | -0.808 | 0.037 |
| SHC (Src homology 2 domain containing) transforming protein 2 | XM_234904 | Shc2 | -1.110 | 0.035 |
| signal peptidase complex subunit 1 homolog (S. cerevisiae) | XM_214276 | Spcs1 | -0.942 | 0.035 |
| Sjogren syndrome antigen B | NM_031119 | Ssb | -0.839 | 0.035 |
| small nuclear ribonucleoprotein 48k (U11/U12) | XM_214453 | Snrnp48 | -0.806 | 0.037 |
| small nuclear ribonucleoprotein D2-like | XM_214847 | Snrpd2l | -1.156 | 0.035 |
| SNF1-like kinase | NM_021693 | Snf1lk | -1.633 | 0.035 |
| solute carrier family 22, member 17 | NM_177421 | Slc22a17 | -0.817 | 0.040 |
| solute carrier family 4, sodium bicarbonate transporter-like, member 11 | AW917183 | Slc4a11 | -1.390 | 0.048 |
| SPARC related modular calcium binding 2 | XM_214777 | Smoc2 | -1.018 | 0.050 |
| SPC24, NDC80 kinetochore complex component, homolog (S. cerevisiae) | XM_343359 | Spc24 | -0.605 | 0.049 |
| spindle and kinetochore associated complex subunit 2 | NM_001009624 | Ska2 | -0.829 | 0.037 |
| stomatin-like 1 | XM_236297 | Stoml1 | -0.804 | 0.044 |
| sulfatase modifying factor 2 | NM_001025125 | Sumf2 | -1.048 | 0.035 |
| suppressor of defective silencing 3 homolog (S. cerevisiae) | XM_341092 | Suds3 | -1.056 | 0.035 |
| SWAP-70 protein | XM_219262 | Swap70 | -0.939 | 0.039 |
| syncollin | NM_139086 | Sycn | -0.832 | 0.035 |
| syntaxin 3 | NM_031124 | Stx3 | -0.988 | 0.048 |
| syntaxin binding protein 5 (tomosyn) | NM_178345 | Stxbp5 | -1.415 | 0.035 |
| TAF15 RNA polymerase II, TATA box binding protein (TBP)-associated factor | XM_237792 | Taf15 | -0.744 | 0.037 |
| TBC1 domain family, member 17 | XM_214940 | Tbc1d17 | -0.832 | 0.035 |
| TGFB-induced factor homeobox 1 | NM_001015020 | Tgif1 | -0.749 | 0.042 |
| thyroid hormone receptor interactor 4 | XM_236360 | Trip4 | -0.771 | 0.037 |
| transducin-like enhancer of split 2 (E(sp1) homolog, Drosophila) | XM_216845 | Tle2 | -1.316 | 0.035 |
| transgelin | NM_031549 | Tagln | -0.977 | 0.035 |
| transmembrane protein 176B | NM_134390 | Tmem176b | -0.898 | 0.035 |
| tudor and KH domain containing | NM_001014038 | Tdrkh | -1.028 | 0.035 |
| tyrosylprotein sulfotransferase 2 | NM_001008508 | Tpst2 | -0.837 | 0.050 |
| ubiquitin-conjugating enzyme E2A, RAD6 homolog (S. cerevisiae) | NM_001013933 | Ube2a | -1.070 | 0.035 |
| ubiquitin-conjugating enzyme E2C | XM_215924 | Ube2c | -0.787 | 0.035 |
| UNC-119 homolog (C. elegans) | NM_017188 | Unc119 | -1.045 | 0.038 |
| UPF3 regulator of nonsense transcripts homolog B (yeast) | XM_233312 | Upf3b | -1.218 | 0.035 |
| vacuolar protein sorting 13 homolog A (S. cerevisiae) | XM_219672 | Vps13a | -0.882 | 0.048 |
| valyl-tRNA synthetase 2, mitochondrial (putative) | NM_213563 | Vars2 | -0.646 | 0.049 |
| vasodilator-stimulated phosphoprotein | XM_341799 | Vasp | -0.735 | 0.050 |
| ventral anterior homeobox 2 | NM_022637 | Vax2 | -0.783 | 0.039 |
| vesicle-associated membrane protein 2 | NM_012663 | Vamp2 | -0.755 | 0.035 |
| v-myc myelocytomatosis viral related oncogene, neuroblastoma derived (avian) | NM_001013096 | Mycn | -1.246 | 0.043 |
| von Willebrand factor A domain containing 1 | NM_001013938 | Vwa1 | -0.934 | 0.035 |
| Vps20-associated 1 homolog (S. cerevisiae) | NM_001025640 | Vta1 | -1.099 | 0.039 |
| WD repeat domain 70 | NM_001013909 | Wdr70 | -0.775 | 0.038 |
| WD repeat domain 74 | XM_574613 | Wdr74 | -0.965 | 0.035 |
| whey acidic protein | NM_053751 | Wap | -2.542 | 0.044 |
| WW domain binding protein 11 | NM_001009661 | Wbp11 | -0.905 | 0.040 |
| YEATS domain containing 2 | CB547405 | Yeats2 | -0.745 | 0.038 |
| zinc finger homeobox 4 | XM_226964 | Zfhx4 | -1.391 | 0.035 |
