# Supplementary material for: In utero exposure to butyl benzyl phthalate induces modifications in the morphology and the gene expression profile of the mammary gland: an experimental study in rats
Source: Environ Health. 2011 Jan 17;10:5. doi: 10.1186/1476-069X-10-5 (PMC3033239; doi:10.1186/1476-069X-10-5)
Supplement: Additional file 2 — Modulated genes by effect of in utero exposure to high dose of BBP. List of known up- and down-regulated genes at 35 days of age in mammary glands of rats exposed prenatally to high dose (500 mg/kg BW) of BBP. For each gene the name, symbol, GeneBank accession number, fold change expression value (in Log2) versus control group, and false discovery rate (FDR) is indicated. [file 1476-069X-10-5-S2.DOC]

**Additional Table 2.**

List of known up- and down-regulated genes at 35 days of age in mammary glands of rats exposed prenatally to high dose (500 mg/kg BW) of BBP. For each gene the name, symbol, GeneBank accession number, fold change expression value (in Log_2_) versus control group, and false discovery rate (FDR) is indicated.

| **Gene Name** | **Symbol** | **Accesion Nº** | ***Log_2_*** | **FDR** |
| --- | --- | --- | --- | --- |
| **Up-modulated:** |  |  |  |  |
| 3-oxoacid CoA transferase 1 | Oxct1 | AY724483 | 1.308 | 0.049 |
| aarF domain containing kinase 1 | Adck1 | AW916730 | 0.600 | 0.042 |
| abhydrolase domain containing 5 | Abhd5 | NM_212524 | 1.125 | 0.042 |
| activin A receptor, type IC | Acvr1c | NM_139090 | 1.600 | 0.040 |
| acyl-Coenzyme A dehydrogenase family, member 11 | Acad11 | XM_236582 | 1.550 | 0.037 |
| ADAM metallopeptidase domain 18 | Adam18 | XM_341456 | 1.026 | 0.036 |
| ADAM metallopeptidase domain 32 | Adam32 | AJ131563 | 0.619 | 0.043 |
| adducin 2 (beta) | Add2 | NM_012491 | 1.147 | 0.036 |
| adenosine deaminase, RNA-specific, B1 | Adarb1 | NM_012894 | 0.655 | 0.046 |
| adenylate cyclase 2 (brain) | Adcy2 | NM_031007 | 1.361 | 0.036 |
| adenylate kinase 1 | Ak1 | NM_024349 | 1.182 | 0.036 |
| ADP-dependent glucokinase | Adpgk | XM_236306 | 0.776 | 0.045 |
| ADP-ribosylation factor-like 5A | Arl5a | NM_053979 | 0.589 | 0.044 |
| ADP-ribosylation factor-like 5B | Arl5b | NM_001015031 | 0.870 | 0.043 |
| ADP-ribosyltransferase 2b | Art2b | AJ297708 | 0.616 | 0.048 |
| adrenergic, alpha-1B-, receptor | Adra1b | NM_016991 | 0.718 | 0.036 |
| adrenergic, beta-3-, receptor | Adrb3 | NM_013108 | 0.952 | 0.041 |
| adrenomedullin | Adm | NM_012715 | 0.879 | 0.046 |
| aldehyde dehydrogenase 6 family, member A1 | Aldh6a1 | NM_031057 | 1.784 | 0.044 |
| aldo-keto reductase family 1, member C-like 2 | Akr1cl2 | NM_001008342 | 1.349 | 0.036 |
| aldolase A, fructose-bisphosphate | Aldoa | NM_012495 | 1.502 | 0.036 |
| aminolevulinate, delta-, synthase 2 | Alas2 | NM_013197 | 0.642 | 0.046 |
| androgen receptor | Ar | NM_012502 | 1.219 | 0.036 |
| angel homolog 1 (Drosophila) | Angel1 | CB544326 | 1.149 | 0.041 |
| angiopoietin 4 | Angpt4 | AW920282 | 2.111 | 0.036 |
| ankyrin repeat domain 5 | Ankrd5 | XM_215854 | 1.333 | 0.037 |
| annexin A1 | Anxa1 | NM_012904 | 0.882 | 0.036 |
| apelin | Apln | NM_031612 | 0.633 | 0.048 |
| arachidonate lipoxygenase 3 | Aloxe3 | XM_213336 | 0.631 | 0.041 |
| arrestin 3, retinal (X-arrestin) | Arr3 | XM_343796 | 0.624 | 0.046 |
| aryl hydrocarbon receptor nuclear translocator-like | Arntl | NM_024362 | 1.694 | 0.041 |
| arylsulfatase family, member I | Arsi | CB545903 | 1.027 | 0.037 |
| aspartoacylase | Aspa | NM_024399 | 2.323 | 0.038 |
| ATP synthase mitochondrial F1 complex assembly factor 1 | Atpaf1 | CB544926 | 0.670 | 0.037 |
| ATP synthase, H+ transporting, mitochondrial F0 complex, subunit C3 (subunit 9) | Atp5g3 | NM_053756 | 0.894 | 0.044 |
| ATPase, Ca++ transporting, cardiac muscle, slow twitch 2 | Atp2a2 | NM_017290 | 0.601 | 0.046 |
| ATPase, class V, type 10D | Atp10d | XM_341209 | 0.718 | 0.037 |
| ATPase, Na+/K+ transporting, alpha 2 polypeptide | Atp1a2 | NM_012505 | 0.995 | 0.037 |
| ATP-binding cassette, sub-family A (ABC1), member 7 | Abca7 | NM_207598 | 0.825 | 0.047 |
| ATP-binding cassette, sub-family A (ABC1), member 8 | Abca8 | XM_221074 | 1.440 | 0.036 |
| ATP-binding cassette, sub-family A (ABC1), member 8a | Abca8a | BF564460 | 1.253 | 0.046 |
| ATP-binding cassette, sub-family D (ALD), member 2 | Abcd2 | NM_033352 | 1.814 | 0.039 |
| ATP-binding cassette, sub-family G (WHITE), member 4 | Abcg4 | XM_236186 | 1.290 | 0.036 |
| BCL2/adenovirus E1B interacting protein 3 | Bnip3 | NM_053420 | 1.712 | 0.036 |
| BCL2-associated athanogene 4 | Bag4 | NM_001025130 | 0.755 | 0.037 |
| bone morphogenetic protein receptor, type IA | Bmpr1a | NM_030849 | 0.823 | 0.037 |
| cache domain containing 1 | Cachd1 | BF567204 | 0.600 | 0.047 |
| calcium binding protein p22 | Chp | NM_024139 | 1.446 | 0.037 |
| carbonic anhydrase 7 | Car7 | XM_226204 | 1.893 | 0.037 |
| carboxylesterase 2-like | Ces2l | NM_133586 | 1.275 | 0.044 |
| carboxylesterase 3 | Ces3 | NM_133295 | 1.991 | 0.037 |
| carboxymethylenebutenolidase homolog (Pseudomonas) | Cmbl | NM_001008770 | 1.553 | 0.036 |
| carboxypeptidase A1 | Cpa1 | NM_016998 | 1.494 | 0.044 |
| carnitine O-octanoyltransferase | Crot | NM_031987 | 0.828 | 0.036 |
| carnitine palmitoyltransferase 1b, muscle | Cpt1b | NM_013200 | 1.103 | 0.036 |
| carnosine dipeptidase 1 (metallopeptidase M20 family) | Cndp1 | NM_001007687 | 0.810 | 0.042 |
| cathepsin B | Ctsb | NM_022597 | 0.731 | 0.037 |
| CCAAT/enhancer binding protein (C/EBP), gamma | Cebpg | X64403 | 0.763 | 0.039 |
| CD151 molecule (Raph blood group) | Cd151 | NM_022523 | 0.723 | 0.042 |
| CD36 molecule (thrombospondin receptor) | Cd36 | NM_031561 | 1.238 | 0.036 |
| CD93 molecule | Cd93 | NM_053383 | 1.083 | 0.036 |
| cell adhesion molecule 3 | Cadm3 | XM_341157 | 0.683 | 0.045 |
| cell death-inducing DFFA-like effector a | Cidea | XM_214551 | 1.664 | 0.038 |
| centrosomal protein 68kDa | Cep68 | XM_214106 | 0.597 | 0.046 |
| centrosomal protein 72kDa | Cep72 | XM_217737 | 0.699 | 0.041 |
| ceroid-lipofuscinosis, neuronal 5 | Cln5 | BF287551 | 1.207 | 0.042 |
| cholinergic receptor, nicotinic, alpha 7 | Chrna7 | NM_012832 | 0.623 | 0.043 |
| chondroitin sulfate N-acetylgalactosaminyltransferase 1 | Csgalnact1 | BF559843 | 1.129 | 0.046 |
| clathrin, light chain (Lcb) | Cltb | NM_053835 | 0.601 | 0.040 |
| coagulation factor VIII, procoagulant component | F8 | NM_183331 | 0.699 | 0.042 |
| coagulation factor XIII, A1 polypeptide | F13a1 | NM_021698 | 0.724 | 0.043 |
| coenzyme Q3 homolog, methyltransferase (S. cerevisiae) | Coq3 | NM_019187 | 0.640 | 0.047 |
| cofilin 2, muscle | Cfl2 | XM_345674 | 0.904 | 0.037 |
| coiled-coil domain containing 80 | Ccdc80 | XM_573284 | 1.129 | 0.036 |
| coiled-coil-helix-coiled-coil-helix domain containing 3 | Chchd3 | XM_238346 | 0.755 | 0.043 |
| collagen, type I, alpha 2 | Col1a2 | NM_053356 | 0.855 | 0.037 |
| colony stimulating factor 1 (macrophage) | Csf1 | NM_023981 | 0.873 | 0.043 |
| complement component 4 binding protein, alpha | C4bpa | NM_012516 | 1.641 | 0.049 |
| complement component 4B (Childo blood group) | C4b | NM_031504 | 0.926 | 0.043 |
| complement factor D (adipsin) | Cfd | XM_343169 | 1.033 | 0.045 |
| COX11 homolog, cytochrome c oxidase assembly protein (yeast) | Cox11 | XM_213433 | 0.776 | 0.037 |
| crystallin, alpha B | Cryab | NM_012935 | 0.877 | 0.036 |
| CTF8, chromosome transmission fidelity factor 8 homolog (S. cerevisiae) | Chtf8 | AI070955 | 0.627 | 0.044 |
| CTP synthase II | Ctps2 | AW917696 | 0.740 | 0.037 |
| C-type lectin domain family 11, member a | Clec11a | NM_001012459 | 0.677 | 0.038 |
| cyclin M4 | Cnnm4 | XM_343554 | 0.809 | 0.036 |
| cyclin-dependent kinase inhibitor 2C (p18, inhibits CDK4) | Cdkn2c | NM_131902 | 1.075 | 0.048 |
| cystatin C | Cst3 | NM_012837 | 0.773 | 0.041 |
| cysteine sulfinic acid decarboxylase | Csad | NM_021750 | 1.003 | 0.036 |
| cysteine/tyrosine-rich 1 | Cyyr1 | NM_001013980 | 0.907 | 0.038 |
| cytochrome c oxidase subunit VIIb | Cox7b | NM_182819 | 0.586 | 0.045 |
| cytochrome c, somatic | Cycs | NM_012839 | 1.148 | 0.044 |
| cytochrome P450, family 1, subfamily b, polypeptide 1 | Cyp1b1 | NM_012940 | 1.720 | 0.037 |
| cytochrome P450, family 2, subfamily J, polypeptide 4 | Cyp2j4 | NM_023025 | 0.830 | 0.042 |
| cytochrome P450, family 27, subfamily b, polypeptide 1 | Cyp27b1 | NM_053763 | 0.674 | 0.037 |
| death-associated protein | Dap | NM_022526 | 0.658 | 0.040 |
| dehydrogenase E1 and transketolase domain containing 1 | Dhtkd1 | NM_001025720 | 1.058 | 0.045 |
| deiodinase, iodothyronine, type I | Dio1 | NM_021653 | 0.656 | 0.050 |
| deleted in malignant brain tumors 1 | Dmbt1 | XM_574572 | 0.844 | 0.037 |
| diazepam binding inhibitor (GABA receptor modulator, acyl-Coenzyme A binding protein) | Dbi | NM_031853 | 1.223 | 0.037 |
| dihydrolipoamide branched chain transacylase E2 | Dbt | AI556228 | 1.285 | 0.044 |
| DnaJ (Hsp40) homolog, subfamily A, member 1 | Dnaja1 | NM_022934 | 0.646 | 0.043 |
| dolichyl pyrophosphate phosphatase 1 | Dolpp1 | AW143320 | 0.703 | 0.044 |
| doublecortin | Dcx | NM_053379 | 1.127 | 0.037 |
| doublecortin-like kinase 1 | Dclk1 | AF030089 | 0.872 | 0.042 |
| doublecortin-like kinase 3 | Dclk3 | BF563650 | 0.842 | 0.036 |
| dual specificity phosphatase 22 | Dusp22 | XM_341523 | 0.832 | 0.037 |
| early B-cell factor 1 | Ebf1 | NM_053820 | 0.765 | 0.048 |
| EF-hand domain family, member D1 | Efhd1 | XM_576608 | 1.128 | 0.041 |
| EH-domain containing 2 | Ehd2 | NM_001024897 | 0.869 | 0.040 |
| electron-transferring-flavoprotein dehydrogenase | Etfdh | NM_198742 | 0.838 | 0.036 |
| endonuclease/exonuclease/phosphatase family domain containing 1 | Eepd1 | NM_001014088 | 1.330 | 0.047 |
| endothelial cell-specific molecule 1 | Esm1 | NM_022604 | 1.334 | 0.036 |
| epithelial membrane protein 1 | Emp1 | NM_012843 | 0.938 | 0.037 |
| eukaryotic translation initiation factor 4E binding protein 1 | Eif4ebp1 | NM_053857 | 0.797 | 0.037 |
| exocyst complex component 6 | Exoc6 | NM_019277 | 0.680 | 0.037 |
| family with sequence similarity 108, member C1 | Fam108c1 | XM_341879 | 0.830 | 0.042 |
| family with sequence similarity 84, member A | Fam84a | XM_234036 | 0.662 | 0.046 |
| fasciculation and elongation protein zeta 2 (zygin II) | Fez2 | AF120111 | 0.783 | 0.047 |
| fibroblast growth factor receptor-like 1 | Fgfrl1 | NM_199114 | 1.128 | 0.048 |
| follistatin | Fst | NM_012561 | 1.374 | 0.037 |
| forkhead box A2 | Foxa2 | NM_012743 | 0.738 | 0.042 |
| four and a half LIM domains 1 | Fhl1 | BC061782 | 1.034 | 0.037 |
| four and a half LIM domains 4 | Fhl4 | NM_001013172 | 0.886 | 0.036 |
| gastric inhibitory polypeptide receptor | Gipr | NM_012714 | 0.828 | 0.041 |
| gephyrin | Gphn | NM_022865 | 0.978 | 0.037 |
| GIPC PDZ domain containing family, member 2 | Gipc2 | XM_342359 | 1.342 | 0.041 |
| glutamate-ammonia ligase (glutamine synthetase) | Glul | M29579 | 0.655 | 0.043 |
| glutathione peroxidase 3 | Gpx3 | D00680 | 1.684 | 0.042 |
| glutathione S-transferase kappa 1 | Gstk1 | NM_181371 | 1.148 | 0.042 |
| glutathione S-transferase theta 1 | Gstt1 | NM_053293 | 1.104 | 0.048 |
| glycogenin 1 | Gyg1 | NM_031043 | 0.598 | 0.043 |
| G-protein coupled receptor 12 | Gpcr12 | NM_030831 | 0.664 | 0.044 |
| growth hormone receptor | Ghr | NM_017094 | 2.086 | 0.045 |
| guanine deaminase | Gda | NM_031776 | 1.341 | 0.044 |
| guanine nucleotide binding protein (G protein), alpha inhibiting 1 | Gnai1 | NM_013145 | 1.404 | 0.037 |
| guanine nucleotide binding protein (G protein), beta 5 | Gnb5 | NM_031770 | 1.143 | 0.037 |
| guanylate cyclase 1, soluble, beta 3 | Gucy1b3 | NM_012769 | 1.465 | 0.037 |
| guanylate cyclase 2F | Gucy2f | NM_053831 | 0.646 | 0.047 |
| haptoglobin | Hp | M34232 | 0.838 | 0.046 |
| HEAT repeat containing 5B | Heatr5b | XM_343000 | 0.619 | 0.042 |
| heat shock protein 12B | Hspa12b | AW918874 | 1.014 | 0.048 |
| heat shock protein 90, alpha (cytosolic), class A member 1 | Hsp90aa1 | NM_175761 | 0.733 | 0.041 |
| HIG1 hypoxia inducible domain family, member 1A | Higd1a | NM_080902 | 0.935 | 0.037 |
| hippocampus abundant transcript-like 1 | Hiatl1 | XM_225093 | 0.704 | 0.042 |
| homeo box A10 | Hoxa10 | XM_347220 | 0.969 | 0.040 |
| homeo box A4 | Hoxa4 | L03557 | 1.030 | 0.037 |
| hydroxyacyl glutathione hydrolase | Hagh | NM_033349 | 0.946 | 0.036 |
| hydroxysteroid (17-beta) dehydrogenase 7 | Hsd17b7 | NM_017235 | 0.623 | 0.037 |
| hydroxysteroid 11-beta dehydrogenase 2 | Hsd11b2 | NM_017081 | 0.941 | 0.036 |
| indolethylamine N-methyltransferase | Inmt | XM_347233 | 1.305 | 0.036 |
| Inositol (myo)-1(or 4)-monophosphatase 1 | Impa1 | NM_032057 | 0.641 | 0.037 |
| insulin-like growth factor 1 | Igf1 | NM_178866 | 0.824 | 0.038 |
| integrin, beta-like 1 | Itgbl1 | NM_001017505 | 1.394 | 0.042 |
| interferon activated gene 204 | Ifi204 | NM_001012029 | 0.973 | 0.036 |
| interleukin 1 receptor, type II | Il1r2 | NM_053953 | 1.446 | 0.037 |
| isovaleryl coenzyme A dehydrogenase | Ivd | NM_012592 | 1.088 | 0.046 |
| jagged 1 | Jag1 | NM_019147 | 0.743 | 0.038 |
| JNK1/MAPK8-associated membrane protein | Jkamp | XM_216728 | 0.753 | 0.037 |
| junctional adhesion molecule 2 | Jam2 | AW918113 | 0.896 | 0.036 |
| kelch-like 29 (Drosophila) | Klhl29 | XM_233955 | 0.869 | 0.041 |
| kidney androgen regulated protein | Kap | NM_052802 | 0.794 | 0.036 |
| Kruppel-like factor 4 (gut) | Klf4 | NM_053713 | 0.938 | 0.040 |
| Kruppel-like factor 9 | Klf9 | AW144489 | 1.061 | 0.044 |
| lactamase, beta 2 | Lactb2 | NM_001024247 | 0.869 | 0.044 |
| laminin, alpha 4 | Lama4 | XM_228209 | 0.921 | 0.037 |
| laminin, gamma 1 | Lamc1 | AW141503 | 0.681 | 0.047 |
| lectin, galactoside-binding, soluble, 3 | Lgals3 | NM_031832 | 0.982 | 0.043 |
| leucine rich repeat containing 8 family, member D | Lrrc8d | NM_001008338 | 1.046 | 0.036 |
| low density lipoprotein receptor-related protein 11 | Lrp11 | XM_238072 | 0.745 | 0.037 |
| low density lipoprotein-related protein 12 | Lrp12 | XM_235261 | 1.158 | 0.037 |
| LYR motif containing 1 | Lyrm1 | XM_344957 | 1.286 | 0.042 |
| Macrophage stimulating 1 (hepatocyte growth factor-like) | Mst1 | NM_024352 | 1.465 | 0.037 |
| MAS-related GPR, member E | Mrgpre | NM_001002288 | 0.587 | 0.044 |
| matrix metallopeptidase 23 | Mmp23 | NM_053606 | 0.979 | 0.036 |
| matrix-remodelling associated 8 | Mxra8 | NM_001007002 | 0.592 | 0.047 |
| MDS1 and EVI1 complex locus | Mecom | XM_226987 | 1.226 | 0.036 |
| mechanistic target of rapamycin (serine/threonine kinase) | Mtor | NM_019906 | 0.702 | 0.037 |
| membrane-bound transcription factor peptidase, site 2 | Mbtps2 | CB548264 | 0.608 | 0.045 |
| microfibrillar associated protein 5 | Mfap5 | XM_342750 | 0.704 | 0.043 |
| microtubule-associated protein tau | Mapt | NM_017212 | 1.200 | 0.048 |
| mitochondrial ribosomal protein L42 | Mrpl42 | XM_216882 | 0.837 | 0.037 |
| mitochondrial ribosomal protein S36 | Mrps36 | XM_215468 | 0.656 | 0.044 |
| mitofusin 2 | Mfn2 | NM_130894 | 0.670 | 0.043 |
| molybdenum cofactor synthesis 2 | Mocs2 | NM_001007633 | 0.823 | 0.042 |
| motile sperm domain containing 1 | Mospd1 | NM_001014107 | 1.334 | 0.037 |
| Mpv17 transgene, kidney disease mutant-like | Mpv17l | XM_340739 | 0.967 | 0.041 |
| mucin 19 | Muc19 | XM_235593 | 0.940 | 0.038 |
| multiple inositol polyphosphate histidine phosphatase 1 | Minpp1 | XM_342044 | 0.659 | 0.043 |
| MyoD family inhibitor domain containing | Mdfic | XM_342643 | 1.271 | 0.037 |
| myosin ID | Myo1d | NM_012983 | 0.822 | 0.045 |
| myosin VIIA and Rab interacting protein | Myrip | BF524140 | 0.652 | 0.037 |
| N-acetyltransferase 1 (arylamine N-acetyltransferase) | Nat1 | NM_053853 | 0.756 | 0.042 |
| NAD(P)H dehydrogenase, quinone 2 | Nqo2 | NM_001004214 | 0.829 | 0.038 |
| natriuretic peptide precursor A | Nppa | NM_012612 | 1.237 | 0.037 |
| NCK-associated protein 1 | Nckap1 | X80029 | 0.780 | 0.037 |
| NEL-like 1 (chicken) | Nell1 | NM_031069 | 0.854 | 0.044 |
| N-ethylmaleimide-sensitive factor attachment protein, gamma | Napg | AW918600 | 0.615 | 0.046 |
| netrin 2-like (chicken) | Ntn2l | XM_343867 | 0.671 | 0.041 |
| neurexin 1 | Nrxn1 | NM_021767 | 0.891 | 0.050 |
| neuroepithelial cell transforming 1 | Net1 | BF555763 | 0.941 | 0.036 |
| neuroplastin | Nptn | NM_019380 | 0.794 | 0.036 |
| neurotrophic tyrosine kinase, receptor, type 2 | Ntrk2 | NM_012731 | 1.637 | 0.042 |
| N-terminal EF-hand calcium binding protein 1 | Necab1 | NM_022302 | 0.611 | 0.043 |
| nuclear factor related to kappa B binding protein | Nfrkb | BM388975 | 0.665 | 0.042 |
| nuclear factor, interleukin 3 regulated | Nfil3 | NM_053727 | 0.719 | 0.048 |
| nuclear receptor subfamily 1, group H, member 3 | Nr1h3 | NM_031627 | 0.726 | 0.042 |
| nuclear receptor subfamily 3, group C, member 1 | Nr3c1 | NM_012576 | 0.807 | 0.043 |
| nudix (nucleoside diphosphate linked moiety X)-type motif 19 | Nudt19 | NM_001004258 | 0.709 | 0.044 |
| odz, odd Oz/ten-m homolog 4 (Drosophila) | Odz4 | XM_218927 | 1.433 | 0.038 |
| olfactory receptor 1077 | Olr1077 | NM_001000941 | 0.652 | 0.040 |
| olfactory receptor 728 | Olr728 | NM_001000363 | 0.605 | 0.044 |
| oxoglutarate dehydrogenase-like | Ogdhl | XM_214261 | 0.766 | 0.046 |
| palmdelphin | Palmd | NM_001025688 | 1.240 | 0.036 |
| paraneoplastic antigen like 5 | Pnma5 | XM_219735 | 0.661 | 0.039 |
| paraoxonase 3 | Pon3 | NM_001004086 | 1.833 | 0.040 |
| PDZ domain containing RING finger 3 | Pdzrn3 | XM_232226 | 1.039 | 0.042 |
| perilipin | Plin | NM_013094 | 1.705 | 0.036 |
| peroxisomal biogenesis factor 7 | Pex7 | AW141902 | 0.894 | 0.037 |
| peroxisomal membrane protein 3 | Pxmp3 | NM_017234 | 0.719 | 0.037 |
| phosphatidylethanolamine N-methyltransferase | Pemt | NM_013003 | 1.110 | 0.036 |
| phosphatidylinositol glycan anchor biosynthesis, class Y | Pigy | NM_001024370 | 1.141 | 0.045 |
| phosphatidylinositol glycan, class K | Pigk | NM_001011953 | 0.648 | 0.042 |
| phosphodiesterase 4D interacting protein | Pde4dip | NM_022382 | 1.535 | 0.037 |
| phosphoenolpyruvate carboxykinase 1 (soluble) | Pck1 | NM_198780 | 1.592 | 0.036 |
| phosphoglucomutase 2 | Pgm2 | XM_214047 | 0.609 | 0.045 |
| phospholipase C, epsilon 1 | Plce1 | NM_053758 | 0.949 | 0.036 |
| phospholipase D1 | Pld1 | AF017251 | 0.592 | 0.043 |
| phospholipid scramblase 4 | Plscr4 | XM_576448 | 0.916 | 0.036 |
| phospholysine phosphohistidine inorganic pyrophosphate phosphatase | Lhpp | NM_001009706 | 0.808 | 0.037 |
| phosphoprotein enriched in astrocytes 15A | Pea15a | NM_001013231 | 0.892 | 0.046 |
| phosphotyrosine interaction domain containing 1 | Pid1 | XM_576601 | 1.662 | 0.038 |
| pleiomorphic adenoma gene-like 1 | Plagl1 | NM_012760 | 1.085 | 0.044 |
| plexin A2 | Plxna2 | XM_223080 | 1.061 | 0.036 |
| polymerase (RNA) II (DNA directed) polypeptide F | Polr2f | NM_031335 | 0.996 | 0.044 |
| potassium channel, subfamily K, member 3 | Kcnk3 | NM_033376 | 1.196 | 0.037 |
| potassium voltage-gated channel, KQT-like subfamily, member 1 | Kcnq1 | NM_032073 | 0.695 | 0.042 |
| potential ligand-binding protein | RY2G5 | X60660 | 0.862 | 0.044 |
| procollagen C-endopeptidase enhancer 2 | PCOLCE2 | BF285037 | 1.956 | 0.036 |
| proprotein convertase subtilisin/kexin type 5 | Pcsk5 | L14933 | 0.826 | 0.037 |
| prostate transmembrane protein, androgen induced 1 | Pmepa1 | XM_230899 | 1.285 | 0.037 |
| proteasome (prosome, macropain) 26S subunit, ATPase, 1 | Psmc1 | NM_057123 | 0.709 | 0.044 |
| proteasome (prosome, macropain) assembly chaperone 2 | Psmg2 | XM_214550 | 0.604 | 0.042 |
| protein disulfide isomerase family A, member 6 | Pdia6 | XM_576132 | 0.859 | 0.036 |
| protein kinase (cAMP-dependent, catalytic) inhibitor alpha | Pkia | BF567508 | 0.788 | 0.038 |
| protein kinase, AMP-activated, gamma 2 non-catalytic subunit | Prkag2 | NM_184051 | 0.605 | 0.047 |
| protein phosphatase 1, regulatory (inhibitor) subunit 1A | Ppp1r1a | NM_022676 | 1.355 | 0.036 |
| protein tyrosine phosphatase 4a1 | Ptp4a1 | NM_031579 | 1.205 | 0.042 |
| protein tyrosine phosphatase, receptor type, B | Ptprb | XM_235156 | 0.850 | 0.037 |
| protein tyrosine phosphatase, receptor type, D | Ptprd | CB547962 | 1.036 | 0.050 |
| protein tyrosine phosphatase, receptor type, O | Ptpro | BF555373 | 0.776 | 0.037 |
| protein tyrosine phosphatase, receptor type, R | Ptprr | NM_053594 | 1.159 | 0.036 |
| pyrophosphatase (inorganic) 1 | Ppa1 | XM_215416 | 0.852 | 0.036 |
| RAB18, member RAS oncogene family | Rab18 | NM_001012468 | 0.936 | 0.045 |
| RAB38, member RAS oncogene family | Rab38 | NM_145774 | 0.888 | 0.044 |
| RAB5B, member RAS oncogene family | Rab5b | BF550034 | 0.626 | 0.043 |
| Rap guanine nucleotide exchange factor (GEF) 2 | Rapgef2 | XM_227301 | 0.914 | 0.046 |
| Ras-like without CAAX 2 | Rit2 | NM_001013060 | 0.868 | 0.038 |
| Ras-related associated with diabetes | Rrad | NM_053338 | 0.931 | 0.037 |
| rearranged L-myc fusion | Rlf | XM_233485 | 0.693 | 0.041 |
| regulator of G-protein signaling 5 | Rgs5 | NM_019341 | 1.028 | 0.044 |
| reticulon 3 | Rtn3 | NM_001009953 | 1.008 | 0.036 |
| reticulon 4 | Rtn4 | NM_031831 | 1.083 | 0.036 |
| retinal pigment epithelium 65 | Rpe65 | NM_053562 | 0.788 | 0.038 |
| retinoic acid receptor responder (tazarotene induced) 2 | Rarres2 | NM_001013427 | 0.872 | 0.043 |
| Rho family GTPase 1 | Rnd1 | NM_001013222 | 1.085 | 0.037 |
| ribonuclease, RNase A family 4 | Rnase4 | NM_020082 | 0.970 | 0.046 |
| ring finger protein 217 | Rnf217 | XM_238063 | 0.700 | 0.037 |
| ring finger protein 8 | Rnf8 | NM_001025727 | 0.682 | 0.042 |
| RNA (guanine-9-) methyltransferase domain containing 1 | Rg9mtd1 | NM_001008337 | 0.649 | 0.038 |
| saccharopine dehydrogenase (putative) | Sccpdh | BC061579 | 0.708 | 0.043 |
| sarcoglycan, epsilon | Sgce | NM_001002023 | 0.733 | 0.040 |
| sarcoglycan, gamma (dystrophin-associated glycoprotein) | Sgcg | NM_001006993 | 1.458 | 0.037 |
| scavenger receptor class A, member 5 (putative) | Scara5 | XM_224299 | 0.756 | 0.037 |
| SEC22 vesicle trafficking protein homolog A (S. cerevisiae) | Sec22a | NM_057147 | 0.776 | 0.045 |
| selectin P | Selp | NM_013114 | 0.871 | 0.044 |
| selenoprotein P, plasma, 1 | Sepp1 | NM_019192 | 0.909 | 0.044 |
| selenoprotein T | Selt | XM_574949 | 0.731 | 0.044 |
| serine (or cysteine) peptidase inhibitor, clade A, member 3N | Serpina3n | NM_031531 | 1.657 | 0.037 |
| serine/threonine kinase 39, STE20/SPS1 homolog (yeast) | Stk39 | NM_019362 | 0.692 | 0.040 |
| serum deprivation response | Sdpr | NM_001007712 | 1.003 | 0.049 |
| small EDRK-rich factor 2 | Serf2 | XM_215807 | 0.662 | 0.040 |
| solute carrier family 2 (facilitated glucose transporter), member 13 | Slc2a13 | NM_133611 | 1.125 | 0.045 |
| solute carrier family 22 (organic anion transporter), member 8 | Slc22a8 | NM_031332 | 0.598 | 0.044 |
| solute carrier family 3, member 1 | Slc3a1 | AI136350 | 0.805 | 0.036 |
| solute carrier family 39 (zinc transporter), member 3 | Slc39a3 | NM_001008356 | 0.737 | 0.036 |
| solute carrier family 46, member 2 | Slc46a2 | CB547809 | 0.593 | 0.044 |
| sorbin and SH3 domain containing 1 | Sorbs1 | AW917667 | 1.407 | 0.037 |
| spectrin, beta, non-erythrocytic 1 | Sptbn1 | AJ242018 | 1.103 | 0.037 |
| spermatogenesis associated 2 | Spata2 | NM_053675 | 0.599 | 0.046 |
| sphingomyelin phosphodiesterase, acid-like 3A | Smpdl3a | NM_001005539 | 1.402 | 0.048 |
| splA/ryanodine receptor domain and SOCS box containing 1 | Spsb1 | XM_233686 | 0.886 | 0.044 |
| SRY (sex determining region Y)-box 17 | Sox17 | XM_232640 | 0.743 | 0.042 |
| ST3 beta-galactoside alpha-2,3-sialyltransferase 3 | St3gal3 | NM_031697 | 0.737 | 0.044 |
| starch binding domain 1 | Stbd1 | NM_001013988 | 0.718 | 0.036 |
| sterol regulatory element binding transcription factor 2 | Srebf2 | BF555125 | 0.682 | 0.047 |
| succinate dehydrogenase complex, subunit C, integral membrane protein | Sdhc | NM_001005534 | 0.948 | 0.037 |
| sulfatase 2 | Sulf2 | XM_230861 | 0.801 | 0.040 |
| sulfotransferase family, cytosolic, 1A, phenol-preferring, member 1 | Sult1a1 | NM_031834 | 1.963 | 0.043 |
| suppression of tumorigenicity 13 | St13 | NM_031122 | 0.587 | 0.043 |
| synapse associated protein 1 | Syap1 | NM_001004253 | 0.794 | 0.045 |
| synaptotagmin XVII | Syt17 | NM_138849 | 0.929 | 0.037 |
| TBC1 domain family, member 14 | Tbc1d14 | NM_001012152 | 0.760 | 0.037 |
| testis specific 10 | Tsga10 | AF092091 | 0.637 | 0.042 |
| tetraspanin 12 | Tspan12 | NM_001015026 | 1.284 | 0.048 |
| tetratricopeptide repeat domain 18 | Ttc18 | XM_341281 | 0.658 | 0.037 |
| thioredoxin 2 | Txn2 | NM_053331 | 0.703 | 0.037 |
| thyroid stimulating hormone receptor | Tshr | NM_012888 | 0.934 | 0.044 |
| transgelin 3 | Tagln3 | NM_031676 | 0.812 | 0.042 |
| trans-golgi network protein | Tgoln1 | NM_138840 | 0.829 | 0.041 |
| transient receptor potential cation channel, subfamily C, member 1 | Trpc1 | NM_053558 | 0.759 | 0.048 |
| transmembrane protein 109 | Tmem109 | NM_001007736 | 0.621 | 0.045 |
| transmembrane protein 45b | Tmem45b | XM_236010 | 0.779 | 0.039 |
| transmembrane protein 70 | Tmem70 | XM_575742 | 1.437 | 0.037 |
| transmembrane protein 85 | Tmem85 | XM_215797 | 0.772 | 0.046 |
| transmembrane protein with EGF-like and two follistatin-like domains 2 | Tmeff2 | XM_343566 | 1.453 | 0.042 |
| triosephosphate isomerase 1 | Tpi1 | NM_022922 | 1.006 | 0.037 |
| tryptophan rich basic protein | Wrb | NM_199373 | 0.774 | 0.038 |
| tubulointerstitial nephritis antigen-like 1 | Tinagl1 | NM_053582 | 0.838 | 0.037 |
| ubiquinol-cytochrome c reductase binding protein | Uqcrb | XM_343225 | 1.068 | 0.045 |
| ubiquitin specific peptidase 14 | Usp14 | NM_001008301 | 0.677 | 0.042 |
| ubiquitination factor E4A | Ube4a | NM_207610 | 0.846 | 0.042 |
| upper zone of growth plate and cartilage matrix associated | Ucma | AA957467 | 0.692 | 0.045 |
| USO1 homolog, vesicle docking protein (yeast) | Uso1 | NM_019379 | 0.652 | 0.040 |
| vacuolar protein sorting 39 homolog (S. cerevisiae) | Vps39 | NM_001012186 | 1.352 | 0.043 |
| very low density lipoprotein receptor | Vldlr | NM_013155 | 0.920 | 0.038 |
| vitamin K epoxide reductase complex, subunit 1-like 1 | Vkorc1l1 | NM_203338 | 1.190 | 0.037 |
| vitronectin | Vtn | NM_019156 | 1.198 | 0.037 |
| von Hippel-Lindau tumor suppressor | Vhl | NM_052801 | 0.616 | 0.037 |
| zinc finger protein 423 | Zfp423 | NM_053583 | 1.633 | 0.037 |
|  |  |  |  |  |
| **Down-modulated:** |  |  |  |  |
| 2',3'-cyclic nucleotide 3' phosphodiesterase | Cnp | NM_012809 | -0.943 | 0.038 |
| 5'-3' exoribonuclease 1 | Xrn1 | XM_217233 | -1.069 | 0.037 |
| 5-oxoprolinase (ATP-hydrolysing) | Oplah | NM_053904 | -0.649 | 0.037 |
| A kinase (PRKA) anchor protein (yotiao) 9 | Akap9 | XM_347223 | -0.676 | 0.046 |
| A kinase (PRKA) anchor protein 8 | Akap8 | XM_343176 | -0.724 | 0.044 |
| achalasia, adrenocortical insufficiency, alacrimia (Allgrove, triple-A) | Aaas | XM_217063 | -0.613 | 0.043 |
| acidic ribosomal phosphoprotein P0 | Arbp | NM_022402 | -0.922 | 0.037 |
| actin related protein 2/3 complex, subunit 5 | Arpc5 | NM_001025717 | -0.704 | 0.038 |
| actin related protein 2/3 complex, subunit 5-like | Arpc5l | XM_216047 | -0.651 | 0.042 |
| activating transcription factor 7 interacting protein | Atf7ip | XM_232488 | -1.661 | 0.036 |
| acyl-CoA synthetase bubblegum family member 1 | Acsbg1 | NM_134389 | -0.668 | 0.042 |
| acyl-Coenzyme A binding domain containing 6 | Acbd6 | NM_001011906 | -0.725 | 0.049 |
| adaptor protein, phosphotyrosine interaction, PH domain and leucine zipper containing 2 | Appl2 | XM_343190 | -0.706 | 0.043 |
| adaptor-related protein complex 3, delta 1 subunit | Ap3d1 | XM_234908 | -1.038 | 0.048 |
| ADP-ribosylation factor GTPase activating protein 1 | Arfgap1 | NM_145090 | -0.661 | 0.047 |
| ADP-ribosylation factor guanine nucleotide-exchange factor 1(brefeldin A-inhibited) | Arfgef1 | XM_232614 | -0.755 | 0.037 |
| advillin | Avil | NM_024401 | -1.196 | 0.043 |
| AF4/FMR2 family, member 3 | Aff3 | XM_343559 | -1.140 | 0.038 |
| alkylglycerone phosphate synthase | Agps | NM_053350 | -0.613 | 0.038 |
| alpha thalassemia/mental retardation syndrome X-linked (RAD54 homolog, S. cerevisiae) | Atrx | BF562870 | -1.012 | 0.037 |
| alpha-2-macroglobulin | A2m | NM_012488 | -1.758 | 0.037 |
| amyloid beta (A4) precursor protein-binding, family B, member 3 | Apbb3 | NM_053957 | -0.732 | 0.038 |
| ankyrin repeat and sterile alpha motif domain containing 3 | Anks3 | NM_001009676 | -0.979 | 0.044 |
| apolipoprotein C-I | Apoc1 | NM_012824 | -1.605 | 0.044 |
| apolipoprotein E | Apoe | NM_138828 | -1.663 | 0.036 |
| apolipoprotein M | Apom | NM_019373 | -0.640 | 0.044 |
| arrestin, beta 2 | Arrb2 | NM_012911 | -1.329 | 0.036 |
| artemin | Artn | NM_053397 | -1.379 | 0.037 |
| AT rich interactive domain 1A (SWI-like) | Arid1a | XM_216340 | -0.688 | 0.041 |
| AT rich interactive domain 5B (Mrf1 like) | Arid5b | XM_228114 | -1.180 | 0.036 |
| ataxin 3 | Atxn3 | NM_021702 | -1.152 | 0.037 |
| ATG16 autophagy related 16-like 1 (S. cerevisiae) | Atg16l1 | BF557691 | -0.722 | 0.037 |
| ATP-binding cassette, sub-family B (MDR/TAP), member 8 | Abcb8 | NM_001007796 | -0.689 | 0.038 |
| baculoviral IAP repeat-containing 3 | Birc3 | NM_023987 | -1.565 | 0.036 |
| basic leucine zipper transcription factor, ATF-like | Batf | XM_216745 | -1.385 | 0.044 |
| BAT2 domain containing 1 | Bat2d1 | XM_341141 | -0.984 | 0.036 |
| B-box and SPRY domain containing | Bspry | NM_022261 | -0.753 | 0.047 |
| B-cell CLL/lymphoma 7C | Bcl7c | XM_215076 | -0.740 | 0.047 |
| B-cell leukemia/lymphoma 2 related protein A1d | Bcl2a1d | NM_133416 | -1.360 | 0.044 |
| B-cell linker | Blnk | NM_001025767 | -1.400 | 0.036 |
| beaded filament structural protein 1 | Bfsp1 | XM_342529 | -0.915 | 0.041 |
| BMS1 homolog, ribosome assembly protein (yeast) | Bms1 | XM_342746 | -0.599 | 0.044 |
| bone marrow stromal cell antigen 2 | Bst2 | NM_198134 | -0.914 | 0.039 |
| branched chain aminotransferase 1, cytosolic | Bcat1 | NM_017253 | -1.242 | 0.037 |
| bridging integrator 2 | Bin2 | NM_001012223 | -2.150 | 0.04 |
| brix domain containing 1 | Bxdc1 | XM_215404 | -0.762 | 0.042 |
| bromodomain and WD repeat domain containing 1 | Brwd1 | XM_221627 | -0.805 | 0.047 |
| bromodomain containing 2 | Brd2 | NM_212495 | -1.374 | 0.036 |
| bromodomain containing 8 | Brd8 | NM_001008509 | -0.763 | 0.047 |
| BUD31 homolog (yeast) | Bud31 | NM_053556 | -0.590 | 0.048 |
| C1q and tumor necrosis factor related protein 3 | C1qtnf3 | XM_226833 | -1.698 | 0.045 |
| calcium homeostasis modulator 2 | Calhm2 | NM_001008306 | -0.700 | 0.037 |
| calpain 1 | Capn1 | U53858 | -1.164 | 0.046 |
| calponin 1, basic, smooth muscle | Cnn1 | NM_031747 | -1.558 | 0.046 |
| carbohydrate (keratan sulfate Gal-6) sulfotransferase 1 | Chst1 | NM_001011955 | -1.155 | 0.036 |
| carboxypeptidase X (M14 family), member 1 | Cpxm1 | XM_215840 | -1.376 | 0.037 |
| carcinoembryonic antigen-related cell adhesion molecule 16 | Ceacam16 | XM_214856 | -0.905 | 0.041 |
| casein alpha s2-like A | Csn1s2a | XM_341197 | -4.336 | 0.05 |
| catenin, beta like 1 | Ctnnbl1 | NM_001024870 | -0.862 | 0.037 |
| cathepsin E | Ctse | NM_012938 | -1.999 | 0.036 |
| CD164 sialomucin-like 2 | Cd164l2 | XM_342935 | -1.390 | 0.036 |
| Cd200 molecule | Cd200 | NM_031518 | -0.948 | 0.044 |
| CD3 molecule, gamma polypeptide | Cd3g | XM_217136 | -3.994 | 0.042 |
| Cd5 molecule | Cd5 | NM_019295 | -4.154 | 0.039 |
| Cd53 molecule | Cd53 | NM_012523 | -2.305 | 0.049 |
| Cd7 molecule | Cd7 | XM_221216 | -0.737 | 0.047 |
| CD83 molecule | Cd83 | XM_341509 | -2.127 | 0.036 |
| CDC45 cell division cycle 45-like (S. cerevisiae) | Cdc45l | XM_213587 | -0.786 | 0.037 |
| CDC5 cell division cycle 5-like (S. pombe) | Cdc5l | NM_053527 | -1.163 | 0.036 |
| CDC-like kinase 3 | Clk3 | NM_134340 | -0.647 | 0.046 |
| CDP-diacylglycerol synthase 1 | Cds1 | NM_031242 | -1.170 | 0.036 |
| centaurin, delta 2 | Centd2 | BC088328 | -0.944 | 0.036 |
| centromere protein F | Cenpf | XM_223060 | -1.603 | 0.045 |
| chemokine (C-C motif) ligand 17 | Ccl17 | NM_057151 | -1.945 | 0.041 |
| chemokine (C-C motif) ligand 19 | Ccl19 | XM_342824 | -2.732 | 0.047 |
| chemokine (C-C motif) ligand 21b (serine) | Ccl21b | NM_001008513 | -2.536 | 0.036 |
| chromodomain helicase DNA binding protein 3 | Chd3 | XM_220602 | -1.295 | 0.041 |
| chromodomain helicase DNA binding protein 7 | Chd7 | XM_232671 | -1.007 | 0.044 |
| cleavage and polyadenylation specific factor 5 | Cpsf5 | XM_214640 | -0.614 | 0.05 |
| cleavage stimulation factor, 3' pre-RNA subunit 2 | Cstf2 | CB546636 | -0.793 | 0.042 |
| cleavage stimulation factor, 3' pre-RNA subunit 2, tau | Cstf2t | AW917690 | -0.825 | 0.036 |
| clusterin | Clu | NM_053021 | -0.611 | 0.047 |
| Cnksr family member 3 | Cnksr3 | NM_001012061 | -0.998 | 0.037 |
| coactosin-like 1 (Dictyostelium) | Cotl1 | XM_341700 | -1.490 | 0.045 |
| coiled-coil domain containing 107 | Ccdc107 | CB544960 | -1.087 | 0.036 |
| coiled-coil domain containing 85B | Ccdc85b | XM_219510 | -0.866 | 0.042 |
| cold inducible RNA binding protein | Cirbp | NM_031147 | -0.661 | 0.043 |
| collagen, type II, alpha 1 | Col2a1 | NM_012929 | -1.611 | 0.037 |
| collagen, type XIV, alpha 1 | Col14a1 | XM_235308 | -0.832 | 0.044 |
| common salivary protein 1 | LOC171161 | NM_133622 | -2.703 | 0.045 |
| copine VI | Cpne6 | XM_240182 | -0.738 | 0.047 |
| corepressor interacting with RBPJ, 1 | Cir1 | NM_001007799 | -0.771 | 0.037 |
| cornichon homolog 4 (Drosophila) | Cnih4 | XM_213960 | -0.885 | 0.037 |
| crystallin, mu | Crym | NM_053955 | -1.154 | 0.036 |
| c-src tyrosine kinase | Csk | NM_001030039 | -0.799 | 0.048 |
| cut-like homeobox 1 | Cux1 | XM_347163 | -1.366 | 0.036 |
| cyclin-dependent kinase inhibitor 1B | Cdkn1b | NM_031762 | -0.745 | 0.047 |
| cysteine-rich, angiogenic inducer, 61 | Cyr61 | NM_031327 | -1.340 | 0.049 |
| cysteine-serine-rich nuclear protein 1 | Csrnp1 | AW916210 | -0.870 | 0.037 |
| D site of albumin promoter (albumin D-box) binding protein | Dbp | NM_012543 | -1.461 | 0.038 |
| dachsous 1 (Drosophila) | Dchs1 | XM_219128 | -1.164 | 0.037 |
| DEK oncogene | Dek | CB545729 | -0.708 | 0.048 |
| DENN/MADD domain containing 2D | Dennd2d | XM_227570 | -1.955 | 0.041 |
| diacylglycerol kinase zeta | Dgkz | NM_031143 | -0.667 | 0.049 |
| dihydrouridine synthase 2-like, SMM1 homolog (S. cerevisiae) | Dus2l | XM_214665 | -0.880 | 0.036 |
| DNA cross-link repair 1C, PSO2 homolog (S. cerevisiae) | Dclre1c | NM_147145 | -1.025 | 0.037 |
| DnaJ (Hsp40) homolog, subfamily C, member 18 | Dnajc18 | NM_001013887 | -0.661 | 0.048 |
| DnaJ (Hsp40) homolog, subfamily C, member 5 | Dnajc5 | NM_024161 | -1.003 | 0.045 |
| docking protein 3 | Dok3 | XM_225170 | -1.759 | 0.036 |
| dolichyl-phosphate mannosyltransferase polypeptide 2, regulatory subunit | Dpm2 | NM_019252 | -0.986 | 0.045 |
| dual adaptor of phosphotyrosine and 3-phosphoinositides | Dapp1 | XM_342348 | -1.252 | 0.047 |
| dual specificity phosphatase 2 | Dusp2 | NM_001012089 | -1.543 | 0.036 |
| dystrobrevin, beta | Dtnb | NM_001012191 | -1.114 | 0.042 |
| dystrophin | Dmd | NM_012698 | -0.810 | 0.042 |
| E1A binding protein p300 | Ep300 | CB547347 | -1.106 | 0.042 |
| E2F transcription factor 5 | E2f5 | XM_574892 | -0.999 | 0.036 |
| early growth response 1 | Egr1 | NM_012551 | -2.258 | 0.044 |
| EF-hand domain (C-terminal) containing 1 | Efhc1 | BF564803 | -1.419 | 0.036 |
| Enah/Vasp-like | Evl | NM_024147 | -1.524 | 0.043 |
| enhancer of zeste homolog 1 (Drosophila) | Ezh1 | XM_220986 | -0.839 | 0.046 |
| epsin 3 | Epn3 | NM_001024791 | -1.964 | 0.042 |
| erythrocyte protein band 4.1-like 3 | Epb4.1l3 | NM_053927 | -1.249 | 0.043 |
| euchromatic histone lysine N-methyltransferase 2 | Ehmt2 | NM_212463 | -1.363 | 0.036 |
| eukaryotic translation initiation factor 1A domain containing | Eif1ad | NM_001008305 | -0.802 | 0.041 |
| eukaryotic translation initiation factor 2 alpha kinase 4 | Eif2ak4 | XM_230462 | -0.868 | 0.036 |
| eukaryotic translation initiation factor 2B, subunit 2 beta | Eif2b2 | NM_032058 | -0.829 | 0.036 |
| eukaryotic translation initiation factor 2C, 1 | Eif2c1 | XM_233544 | -0.860 | 0.04 |
| eukaryotic translation initiation factor 3, subunit G | Eif3g | NM_001013095 | -0.835 | 0.037 |
| exosome component 8 | Exosc8 | XM_215566 | -0.915 | 0.041 |
| ezrin | Ezr | NM_019357 | -1.454 | 0.038 |
| family with sequence similarity 113, member A | Fam113a | XM_215835 | -1.014 | 0.037 |
| family with sequence similarity 117, member B | Fam117b | XM_343576 | -0.634 | 0.038 |
| family with sequence similarity 189, member B | Fam189b | XM_227412 | -1.066 | 0.043 |
| Fanconi anemia, complementation group C | Fancc | NM_012557 | -0.709 | 0.037 |
| FAT tumor suppressor homolog 1 (Drosophila) | Fat1 | NM_031819 | -0.643 | 0.047 |
| fatty acid amide hydrolase | Faah | NM_024132 | -1.006 | 0.047 |
| F-box and leucine-rich repeat protein 19 | Fbxl19 | XM_219356 | -1.254 | 0.045 |
| Fc fragment of IgG, low affinity IIb, receptor (CD32) | Fcgr2b | NM_175756 | -1.397 | 0.045 |
| fetal Alzheimer antigen | Falz | XM_221050 | -0.740 | 0.037 |
| fibroblast growth factor (acidic) intracellular binding protein | Fibp | NM_172334 | -0.830 | 0.042 |
| fibroblast growth factor receptor 4 | Fgfr4 | M91599 | -0.816 | 0.037 |
| ficolin (collagen/fibrinogen domain containing) 1 | Fcn1 | NM_031348 | -0.735 | 0.047 |
| FK506 binding protein 1a | Fkbp1a | NM_013102 | -0.682 | 0.037 |
| forkhead box P1 | Foxp1 | XM_216227 | -0.934 | 0.036 |
| four and a half LIM domains 2 | Fhl2 | NM_031677 | -0.925 | 0.042 |
| G protein-coupled receptor 160 | Gpr160 | NM_001025147 | -1.015 | 0.037 |
| gamma-glutamyl hydrolase | Ggh | NM_012960 | -0.775 | 0.049 |
| Gardner-Rasheed feline sarcoma viral (v-fgr) oncogene homolog | Fgr | NM_024145 | -0.748 | 0.048 |
| general transcription factor IIH, polypeptide 2 | Gtf2h2 | XM_215466 | -0.739 | 0.048 |
| glioma tumor suppressor candidate region gene 2 | Gltscr2 | NM_207591 | -0.634 | 0.042 |
| glucosamine (UDP-N-acetyl)-2-epimerase/N-acetylmannosamine kinase | Gne | NM_053765 | -1.313 | 0.036 |
| glucosamine-phosphate N-acetyltransferase 1 | Gnpnat1 | AW915606 | -0.724 | 0.037 |
| glutamate decarboxylase 1 | Gad1 | NM_017007 | -2.717 | 0.037 |
| glutamate receptor, ionotrophic, AMPA 3 | Gria3 | NM_032990 | -1.347 | 0.043 |
| glycosylation dependent cell adhesion molecule 1 | Glycam1 | NM_012794 | -2.402 | 0.037 |
| glycosylphosphatidylinositol specific phospholipase D1 | Gpld1 | XM_214480 | -1.160 | 0.043 |
| golgi associated, gamma adaptin ear containing, ARF binding protein 3 | Gga3 | CB606450 | -0.590 | 0.048 |
| golgi phosphoprotein 2 | Golph2 | XM_225147 | -1.363 | 0.036 |
| G-protein signaling modulator 3 (AGS3-like, C. elegans) | Gpsm3 | NM_001003974 | -1.056 | 0.036 |
| GRAM domain containing 1A | Gramd1a | NM_001014160 | -1.152 | 0.036 |
| growth arrest and DNA-damage-inducible, beta | Gadd45b | NM_001008321 | -1.088 | 0.036 |
| growth arrest specific 5 | Gas5 | U77829 | -1.396 | 0.037 |
| growth arrest specific 6 | Gas6 | BC070881 | -1.178 | 0.036 |
| guanine nucleotide binding protein (G protein), alpha z polypeptide | Gnaz | NM_013189 | -0.958 | 0.046 |
| guanine nucleotide binding protein, alpha 15 | Gna15 | NM_053542 | -0.998 | 0.036 |
| guanine nucleotide binding protein-like 1 | Gnl1 | NM_212500 | -1.425 | 0.037 |
| H2A histone family, member X | H2afx | XM_576399 | -1.595 | 0.037 |
| HCCA2 protein | Hcca2 | XM_574587 | -0.785 | 0.036 |
| headcase homolog (Drosophila) | Heca | XM_218660 | -0.996 | 0.036 |
| heterogeneous nuclear ribonucleoprotein H1 | Hnrph1 | NM_080896 | -0.928 | 0.048 |
| heterogeneous nuclear ribonucleoprotein U-like 1 | Hnrnpul1 | XM_341807 | -0.832 | 0.037 |
| high mobility group box 1 | Hmgb1 | NM_012963 | -0.839 | 0.046 |
| HIRA interacting protein 3 | Hirip3 | NM_001025725 | -0.841 | 0.037 |
| histidine acid phosphatase domain containing 2A | Hisppd2a | XM_230503 | -0.629 | 0.046 |
| histone acetyltransferase 1 | Hat1 | NM_001009657 | -0.867 | 0.043 |
| histone deacetylase 2 | Hdac2 | XM_342149 | -0.851 | 0.037 |
| HLA-B associated transcript 1 | Bat1 | NM_133300 | -1.190 | 0.037 |
| HMG box domain containing 4 | Hmgxb4 | XM_226315 | -0.803 | 0.046 |
| homeodomain interacting protein kinase 4 | Hipk4 | NM_001024776 | -1.315 | 0.048 |
| homer homolog 1 (Drosophila) | Homer1 | NM_031707 | -1.099 | 0.047 |
| hyaluronoglucosaminidase 2 | Hyal2 | NM_172040 | -0.786 | 0.046 |
| hydroxyacylglutathione hydrolase-like | Haghl | NM_001013114 | -1.006 | 0.037 |
| hydroxysteroid (17-beta) dehydrogenase 11 | Hsd17b11 | NM_001004209 | -0.652 | 0.047 |
| hypothetical protein LOC619574 | LOC619574 | BC079126 | -0.608 | 0.044 |
| hypothetical protein LOC691259 | LOC691259 | XM_213308 | -0.712 | 0.038 |
| immediate early response 3 | Ier3 | NM_212505 | -0.969 | 0.043 |
| immunoglobulin superfamily, member 8 | Igsf8 | NM_001014787 | -1.210 | 0.036 |
| IMP (inosine monophosphate) dehydrogenase 2 | Impdh2 | NM_199099 | -1.327 | 0.036 |
| inhibitor of growth family, member 1 | Ing1 | BC086336 | -0.893 | 0.036 |
| inositol 1,4,5-triphosphate receptor, type 3 | Itpr3 | NM_013138 | -1.109 | 0.036 |
| inositol hexakisphosphate kinase 2 | Ip6k2 | XM_576471 | -0.808 | 0.044 |
| inositol polyphosphate-5-phosphatase B | Inpp5b | XM_342909 | -0.710 | 0.042 |
| integral membrane protein 2C | Itm2c | NM_001009674 | -0.801 | 0.039 |
| integrator complex subunit 10 | Ints10 | XM_214334 | -0.608 | 0.048 |
| integrin, beta 6 | Itgb6 | NM_001004263 | -2.267 | 0.049 |
| integrin-linked kinase | Ilk | NM_133409 | -0.648 | 0.047 |
| interferon induced transmembrane protein 1 | Ifitm1 | XM_215117 | -1.141 | 0.037 |
| interferon regulatory factor 3 | Irf3 | NM_001006969 | -0.614 | 0.045 |
| interferon regulatory factor 6 | Irf6 | XM_344194 | -1.806 | 0.037 |
| interleukin 1 beta | Il1b | NM_031512 | -2.225 | 0.036 |
| interleukin 18 | Il18 | NM_019165 | -2.231 | 0.037 |
| interleukin 2 receptor, beta | Il2rb | NM_013195 | -1.431 | 0.044 |
| IQ motif containing B1 | Iqcb1 | XM_221420 | -1.104 | 0.043 |
| isoleucyl-tRNA synthetase | Iars | XM_225196 | -0.980 | 0.047 |
| Janus kinase 2 | Jak2 | NM_031514 | -0.684 | 0.046 |
| Jun oncogene | Jun | X17215 | -0.656 | 0.047 |
| junctional adhesion molecule 3 | Jam3 | NM_001004269 | -0.967 | 0.038 |
| karyopherin alpha 2 | Kpna2 | NM_053483 | -1.099 | 0.04 |
| katanin p60 (ATPase-containing) subunit A1 | Katna1 | NM_001004217 | -0.705 | 0.044 |
| kelch-like 5 (Drosophila) | Klhl5 | XM_223418 | -1.010 | 0.037 |
| kelch-like 8 (Drosophila) | Klhl8 | XM_213995 | -1.002 | 0.042 |
| kinectin 1 | Ktn1 | XM_341305 | -0.802 | 0.046 |
| kinesin family member 20B | Kif20b | CB545413 | -0.859 | 0.044 |
| KRAB-A domain containing 1 | Krba1 | XM_342681 | -1.331 | 0.037 |
| leucine rich repeat containing 42 | Lrrc42 | NM_001025653 | -1.078 | 0.043 |
| leukocyte receptor cluster (LRC) member 1 | Leng1 | XM_214797 | -1.355 | 0.036 |
| LIM domain binding 1 | Ldb1 | XM_219948 | -0.921 | 0.037 |
| lin-37 homolog (C. elegans) | Lin37 | XM_214898 | -0.755 | 0.043 |
| linker for activation of T cells | Lat | NM_030853 | -1.861 | 0.048 |
| lymphocyte cytosolic protein 1 | Lcp1 | NM_001012044 | -1.453 | 0.048 |
| lymphocyte-specific protein tyrosine kinase | Lck | XM_232763 | -2.821 | 0.048 |
| lymphotoxin beta (TNF superfamily, member 3) | Ltb | NM_212507 | -3.013 | 0.041 |
| lysophosphatidic acid receptor 1 | Lpar1 | NM_053936 | -1.119 | 0.046 |
| lysosomal protein transmembrane 5 | Laptm5 | NM_053538 | -1.657 | 0.042 |
| major facilitator superfamily domain containing 4 | Mfsd4 | XM_573448 | -1.938 | 0.036 |
| major facilitator superfamily domain containing 6 | Mfsd6 | BG665267 | -0.941 | 0.037 |
| mammary cancer associated protein RMT-1 | Rmt1 | NM_145088 | -0.902 | 0.036 |
| mannose-6-phosphate receptor, cation dependent | M6pr | NM_001007700 | -0.598 | 0.042 |
| mannosidase, alpha, class 2A, member 1 | Man2a1 | H32368 | -0.964 | 0.036 |
| MAP-kinase activating death domain | Madd | NM_053585 | -1.344 | 0.037 |
| matrix metallopeptidase 2 | Mmp2 | U65656 | -0.680 | 0.037 |
| Mdm4 p53 binding protein homolog (mouse) | Mdm4 | NM_001012026 | -1.228 | 0.036 |
| mediator complex subunit 13 | Med13 | XM_220813 | -1.163 | 0.036 |
| Mediterranean fever | Mefv | NM_031634 | -1.035 | 0.037 |
| membrane bound O-acyltransferase domain containing 1 | Mboat1 | XM_574019 | -1.119 | 0.048 |
| menage a trois homolog 1, cyclin H assembly factor (Xenopus laevis) | Mnat1 | NM_153472 | -0.786 | 0.042 |
| metal response element binding transcription factor 2 | Mtf2 | XM_341180 | -0.851 | 0.041 |
| metastasis associated 1 | Mta1 | NM_022588 | -0.756 | 0.041 |
| methylenetetrahydrofolate dehydrogenase (NADP+ dependent) 1-like | Mthfd1l | XM_341750 | -1.869 | 0.036 |
| microtubule associated monoxygenase, calponin and LIM domain containing 1 | Mical1 | XM_215424 | -0.784 | 0.047 |
| microtubule-actin crosslinking factor 1 | Macf1 | XM_342906 | -0.899 | 0.037 |
| microtubule-associated protein 2 | Map2 | U30938 | -1.584 | 0.042 |
| microtubule-associated protein 7 | Map7 | XM_214968 | -0.943 | 0.047 |
| milk fat globule-EGF factor 8 protein | Mfge8 | NM_012811 | -1.370 | 0.037 |
| minichromosome maintenance deficient 7 (S. cerevisiae) | Mcm7 | NM_001004203 | -0.932 | 0.037 |
| mitochondrial ribosomal protein L9 | mrpl9 | NM_001007696 | -0.629 | 0.046 |
| mitogen activated protein kinase kinase kinase 2 | Map3k2 | XM_226073 | -0.772 | 0.042 |
| mitogen-activated protein kinase kinase 1 interacting protein 1 | Map2k1ip1 | NM_001008375 | -0.586 | 0.048 |
| mitogen-activated protein kinase kinase kinase 11 | Map3k11 | NM_001013150 | -1.023 | 0.041 |
| M-phase phosphoprotein 10 (U3 small nucleolar ribonucleoprotein) | Mphosph10 | XM_238166 | -1.218 | 0.036 |
| myb-like, SWIRM and MPN domains 1 | Mysm1 | XM_216460 | -0.756 | 0.037 |
| myeloid/lymphoid or mixed-lineage leukemia (trithorax homolog, Drosophila); translocated to, 10 | Mllt10 | NM_001012162 | -1.241 | 0.036 |
| myeloid/lymphoid or mixed-lineage leukemia 1 | Mll1 | XM_236194 | -1.361 | 0.039 |
| myosin VC | Myo5c | CB544339 | -2.213 | 0.036 |
| MYST histone acetyltransferase 2 | Myst2 | NM_181081 | -0.599 | 0.043 |
| N-6 adenine-specific DNA methyltransferase 2 (putative) | N6amt2 | XM_214202 | -0.993 | 0.037 |
| nascent-polypeptide-associated complex alpha polypeptide | Naca | XM_213821 | -0.710 | 0.038 |
| NCK adaptor protein 2 | Nck2 | XM_237115 | -0.872 | 0.037 |
| NCK associated protein 1 like | Nckap1l | XM_235709 | -0.751 | 0.045 |
| neuroguidin, EIF4E binding protein | Ngdn | XM_224181 | -0.914 | 0.037 |
| neutrophil cytosolic factor 4 | Ncf4 | BF565335 | -1.603 | 0.041 |
| nuclear mitotic apparatus protein 1 | Numa1 | XM_218972 | -0.809 | 0.037 |
| nuclear receptor binding SET domain protein 1 | Nsd1 | XM_225168 | -0.604 | 0.046 |
| nuclear receptor subfamily 2, group F, member 6 | Nr2f6 | NM_139113 | -0.668 | 0.042 |
| nuclear transcription factor, X-box binding 1 | Nfx1 | NM_001024784 | -1.074 | 0.046 |
| nuclear transcription factor-Y beta | Nfyb | NM_031553 | -0.718 | 0.037 |
| nucleolar and coiled-body phosphoprotein 1 | Nolc1 | NM_022869 | -1.031 | 0.036 |
| nucleoporin 107 | Nup107 | NM_053830 | -1.148 | 0.036 |
| nucleoporin 210 | Nup210 | NM_053322 | -1.611 | 0.047 |
| oral-facial-digital syndrome 1 gene homolog (human) | Ofd1 | XM_217615 | -0.748 | 0.04 |
| oxidative stress induced growth inhibitor 1 | Osgin1 | NM_138504 | -1.625 | 0.047 |
| pan hematopoietic expression | Phemx | BC088266 | -2.333 | 0.045 |
| PAP associated domain containing 5 | Papd5 | XM_226334 | -0.722 | 0.05 |
| par-3 (partitioning defective 3) homolog (C. elegans) | Pard3 | NM_031235 | -1.156 | 0.043 |
| parvin, alpha | Parva | NM_020656 | -0.749 | 0.044 |
| PC4 and SFRS1 interacting protein 1 | Psip1 | NM_175765 | -0.989 | 0.036 |
| peptidylprolyl isomerase (cyclophilin)-like 1 | Ppil1 | XM_228046 | -0.843 | 0.038 |
| peptidylprolyl isomerase C | Ppic | NM_001004215 | -0.672 | 0.037 |
| pescadillo homolog 1, containing BRCT domain (zebrafish) | Pes1 | CB547305 | -0.658 | 0.041 |
| PHD finger protein 14 | Phf14 | XM_575385 | -0.700 | 0.044 |
| PHD finger protein 19 | Phf19 | XM_580016 | -0.601 | 0.041 |
| phosphatidylcholine transfer protein | Pctp | NM_017225 | -0.814 | 0.04 |
| phosphatidylethanolamine binding protein 1 | Pebp1 | NM_017236 | -1.004 | 0.037 |
| phosphatidylinositol-3,4,5-trisphosphate-dependent Rac exchange factor 1 | Prex1 | XM_230873 | -1.293 | 0.037 |
| phosphodiesterase 4B, cAMP specific | Pde4b | NM_017031 | -1.012 | 0.037 |
| phosphoenolpyruvate carboxykinase 2 (mitochondrial) | Pck2 | XM_341319 | -1.116 | 0.043 |
| phosphofurin acidic cluster sorting protein 1 | Pacs1 | NM_134406 | -0.644 | 0.047 |
| phospholipase C, gamma 1 | Plcg1 | NM_013187 | -0.632 | 0.049 |
| phospholipase C, gamma 2 | Plcg2 | NM_017168 | -0.798 | 0.042 |
| phospholipase D family, member 3 | Pld3 | NM_001012167 | -0.612 | 0.037 |
| phospholipid scramblase 1 | Plscr1 | NM_057194 | -1.209 | 0.036 |
| phospholipid scramblase 2 | Plscr2 | NM_001014094 | -1.178 | 0.036 |
| phosphoribosyl pyrophosphate synthetase-associated protein 1 | Prpsap1 | NM_022545 | -1.205 | 0.036 |
| phosphorylase kinase, gamma 2 (testis) | Phkg2 | NM_080584 | -0.747 | 0.037 |
| poly (ADP-ribose) polymerase family, member 8 | Parp8 | XM_215491 | -0.948 | 0.037 |
| poly(A) binding protein, cytoplasmic 1 | Pabpc1 | NM_134353 | -0.772 | 0.042 |
| polymerase (DNA directed), epsilon | Pole | XM_222255 | -0.909 | 0.037 |
| polymerase (DNA directed), gamma 2, accessory subunit | Polg2 | XM_221047 | -0.843 | 0.042 |
| polymerase (DNA-directed), delta 3, accessory subunit | Pold3 | NM_001024750 | -0.911 | 0.041 |
| polymerase (RNA) I polypeptide C | Polr1c | NM_001008330 | -0.716 | 0.048 |
| polymerase (RNA) II (DNA directed) polypeptide H-like | Polr2hl | XM_213574 | -0.734 | 0.047 |
| polynucleotide kinase 3'-phosphatase | Pnkp | NM_001004259 | -1.006 | 0.036 |
| potassium channel tetramerisation domain containing 1 | Kctd1 | XM_214617 | -1.525 | 0.036 |
| potassium intermediate/small conductance calcium-activated channel, subfamily N, member 4 | Kcnn4 | NM_023021 | -1.895 | 0.036 |
| POU class 3 homeobox 3 | Pou3f3 | NM_138837 | -1.562 | 0.037 |
| proteasome (prosome, macropain) subunit, beta type 8 (large multifunctional peptidase 7) | Psmb8 | NM_080767 | -1.051 | 0.036 |
| proteasome (prosome, macropain) subunit, beta type 9 (large multifunctional peptidase 2) | Psmb9 | NM_012708 | -0.930 | 0.042 |
| proteasome maturation protein | Pomp | XM_213700 | -1.025 | 0.036 |
| protein arginine methyltransferase 1 | Prmt1 | NM_024363 | -0.915 | 0.044 |
| protein arginine methyltransferase 7 | Prmt7 | NM_001014153 | -0.756 | 0.043 |
| protein interacting with PRKCA 1 | Pick1 | NM_053460 | -0.981 | 0.042 |
| protein kinase C, delta | Prkcd | NM_133307 | -0.888 | 0.046 |
| protein kinase C, theta | Prkcq | XM_341553 | -1.351 | 0.049 |
| protein kinase D2 | Prkd2 | NM_001013895 | -0.717 | 0.037 |
| protein kinase, cAMP dependent regulatory, type I, alpha | Prkar1a | CF110967 | -0.683 | 0.043 |
| protein O-linked mannose beta1,2-N-acetylglucosaminyltransferase | Pomgnt1 | NM_001007747 | -0.805 | 0.036 |
| protein regulator of cytokinesis 1 | Prc1 | XM_218820 | -1.582 | 0.046 |
| protein tyrosine phosphatase, non-receptor type 13 | Ptpn13 | XM_573554 | -0.714 | 0.042 |
| protein tyrosine phosphatase, non-receptor type 22 (lymphoid) | Ptpn22 | XM_215658 | -3.063 | 0.047 |
| protein tyrosine phosphatase, non-receptor type 6 | Ptpn6 | NM_053908 | -1.566 | 0.036 |
| protein tyrosine phosphatase, non-receptor type 7 | Ptpn7 | NM_145683 | -1.519 | 0.044 |
| NA (Prtpc-predicted) | NA | NA | -2.549 | 0.048 |
| prothymosin alpha | Ptma | NM_021740 | -0.907 | 0.042 |
| pumilio homolog 1 (Drosophila) | Pum1 | XM_342928 | -0.669 | 0.042 |
| R3H domain and coiled-coil containing 1 | R3hcc1 | XM_341347 | -0.933 | 0.047 |
| RAB, member of RAS oncogene family-like 2B | Rabl2b | NM_001013221 | -0.665 | 0.042 |
| RAB25, member RAS oncogene family | Rab25 | XM_227404 | -1.913 | 0.04 |
| RAB4B, member RAS oncogene family | Rab4b | NM_017355 | -0.940 | 0.04 |
| RAD21 homolog (S. pombe) | Rad21 | NM_001025701 | -0.767 | 0.047 |
| RAD52 homolog (S. cerevisiae) | Rad52 | XM_216230 | -1.102 | 0.036 |
| Ral GEF with PH domain and SH3 binding motif 2 | Ralgps2 | XM_222773 | -1.525 | 0.036 |
| RALBP1 associated Eps domain containing 1 | Reps1 | XM_214954 | -1.212 | 0.036 |
| RanBP-type and C3HC4-type zinc finger containing 1 | Rbck1 | NM_021764 | -1.165 | 0.037 |
| Rap guanine nucleotide exchange factor (GEF) 6 | Rapgef6 | XM_220428 | -0.685 | 0.043 |
| RAP1 interacting factor homolog (yeast) | Rif1 | BF287849 | -1.128 | 0.037 |
| Ras and Rab interactor 2 | Rin2 | XM_230647 | -1.037 | 0.036 |
| Ras homolog enriched in brain like 1 | Rhebl1 | NM_182825 | -1.837 | 0.037 |
| regenerating islet-derived 3 beta | Reg3b | NM_053289 | -2.181 | 0.036 |
| regenerating islet-derived 3 gamma | Reg3g | NM_173097 | -0.898 | 0.042 |
| regulator of G-protein signaling 1 | Rgs1 | U77698 | -1.788 | 0.037 |
| regulator of G-protein signaling 10 | Rgs10 | XM_341936 | -1.166 | 0.036 |
| regulatory factor X, 7 | Rfx7 | XM_236392 | -1.097 | 0.036 |
| remodeling and spacing factor 1 | Rsf1 | XM_218939 | -0.799 | 0.037 |
| renin binding protein | Renbp | NM_031095 | -1.238 | 0.047 |
| ret proto-oncogene | Ret | NM_012643 | -1.901 | 0.042 |
| reticulocalbin 1, EF-hand calcium binding domain | Rcn1 | XM_342481 | -1.200 | 0.037 |
| reticulocalbin 3, EF-hand calcium binding domain | Rcn3 | NM_001008694 | -1.094 | 0.041 |
| retinoblastoma binding protein 6 | Rbbp6 | XM_219296 | -0.885 | 0.036 |
| retinoic acid receptor responder (tazarotene induced) 1 | Rarres1 | NM_001014790 | -0.884 | 0.039 |
| REV1 homolog (S. cerevisiae) | Rev1 | XM_237071 | -0.948 | 0.038 |
| Rho GTPase activating protein 11A | Arhgap11a | XM_230459 | -1.216 | 0.036 |
| rhomboid 5 homolog 1 (Drosophila) | Rhbdf1 | NM_001030034 | -0.617 | 0.045 |
| rhophilin, Rho GTPase binding protein 1 | Rhpn1 | BM390495 | -0.602 | 0.045 |
| ribosomal protein L19 | Rpl19 | NM_031103 | -0.815 | 0.042 |
| ribosomal protein L24 | Rpl24 | NM_022515 | -1.114 | 0.036 |
| ribosomal protein L27 | Rpl27 | NM_022514 | -0.948 | 0.037 |
| ribosomal protein L28 | Rpl28 | X52619 | -0.630 | 0.047 |
| ribosomal protein L30 | Rpl30 | NM_022699 | -1.281 | 0.036 |
| ribosomal protein L36 | Rpl36 | NM_022504 | -0.717 | 0.046 |
| ribosomal protein L37 | Rpl37 | NM_031106 | -0.730 | 0.048 |
| ribosomal protein L38 | Rpl38 | X57007 | -0.724 | 0.046 |
| ribosomal protein L4 | Rpl4 | NM_022510 | -0.918 | 0.036 |
| ribosomal protein L41 | Rpl41 | NM_139083 | -1.416 | 0.036 |
| ribosomal protein S10 | Rps10 | NM_031109 | -0.609 | 0.044 |
| ribosomal protein S11 | Rps11 | NM_031110 | -0.757 | 0.037 |
| ribosomal protein S15 | Rps15 | NM_017151 | -0.967 | 0.037 |
| ribosomal protein S19 | Rps19 | XM_574391 | -1.192 | 0.036 |
| ribosomal protein S21 | Rps21 | NM_031111 | -1.167 | 0.036 |
| ribosomal protein s25 | Rps25 | NM_001005528 | -0.783 | 0.037 |
| ribosomal protein S3a | Rps3a | NM_017153 | -1.050 | 0.036 |
| ribosomal protein S6 kinase polypeptide 1 | Rps6ka1 | NM_031107 | -1.387 | 0.036 |
| ring finger protein 145 | Rnf145 | XM_213277 | -0.881 | 0.036 |
| ring finger protein 149 | Rnf149 | XM_343561 | -0.740 | 0.041 |
| RNA binding motif protein 5 | Rbm5 | XM_217263 | -0.592 | 0.047 |
| RNA binding motif protein, X chromosome retrogene-like | Rbmxrtl | XM_226369 | -0.919 | 0.042 |
| RNA binding motif protein, X-linked 2 | Rbmx2 | XM_346355 | -0.916 | 0.036 |
| RT1 class II, locus Db1 | RT1-Db1 | AJ003232 | -1.283 | 0.037 |
| S100 calcium binding protein A6 | S100a6 | NM_053485 | -1.642 | 0.037 |
| SAP domain containing ribonucleoprotein | Sarnp | XM_343147 | -0.843 | 0.037 |
| SAPS domain family, member 1 | Saps1 | XM_341781 | -0.643 | 0.043 |
| sarcoma antigen NY-SAR-48 | Ny-sar-48 | NM_001024971 | -0.678 | 0.04 |
| scavenger receptor class F, member 2 | Scarf2 | XM_237825 | -0.844 | 0.036 |
| secreted frizzled-related protein 2 | Sfrp2 | XM_227314 | -1.398 | 0.042 |
| secretory leukocyte peptidase inhibitor | Slpi | NM_053372 | -1.997 | 0.037 |
| selenoprotein W, 1 | Sepw1 | NM_013027 | -0.889 | 0.036 |
| serine (or cysteine) proteinase inhibitor, clade B, member 1a | Serpinb1a | XM_214459 | -1.209 | 0.037 |
| serine/threonine kinase 38 | Stk38 | NM_001015025 | -0.609 | 0.043 |
| serine/threonine protein kinase MST4 | Mst4 | CB547252 | -1.223 | 0.036 |
| serologically defined colon cancer antigen 1, pseudogene 1 | Sdccag1-ps1 | XM_216724 | -0.826 | 0.036 |
| SET domain containing 2 | Setd2 | XM_236648 | -0.631 | 0.042 |
| seven in absentia 2 | Siah2 | NM_134457 | -0.641 | 0.046 |
| SH3 and multiple ankyrin repeat domains 2 | Shank2 | NM_133441 | -0.679 | 0.043 |
| SH3-domain GRB2-like 3 | Sh3gl3 | NM_031238 | -1.028 | 0.037 |
| Sh3kbp1 binding protein 1 | Shkbp1 | XM_214873 | -0.956 | 0.037 |
| SHC (Src homology 2 domain containing) transforming protein 2 | Shc2 | XM_234904 | -1.200 | 0.038 |
| siah binding protein 1; FBP interacting repressor; pyrimidine tract binding splicing factor; Ro ribonucleoprotein-binding protein 1 | Siahbp1 | XM_343268 | -0.715 | 0.042 |
| signal transducer and activator of transcription 2 | Stat2 | NM_001011905 | -0.844 | 0.043 |
| signal transducer and activator of transcription 4 | Stat4 | NM_001012226 | -1.773 | 0.046 |
| signal transducing adaptor family member 1 | Stap1 | NM_001025115 | -1.290 | 0.041 |
| Sjogren syndrome antigen B | Ssb | NM_031119 | -1.107 | 0.036 |
| small nuclear ribonucleoprotein 48k (U11/U12) | Snrnp48 | XM_214453 | -0.887 | 0.047 |
| small nuclear ribonucleoprotein D2-like | Snrpd2l | XM_214847 | -1.345 | 0.036 |
| small nuclear ribonucleoprotein polypeptide F | Snrpf | XM_345814 | -0.803 | 0.043 |
| sodium channel, nonvoltage-gated, type I, beta | Scnn1b | NM_012648 | -1.421 | 0.036 |
| solute carrier family 2 (facilitated glucose transporter), member 1 | Slc2a1 | NM_138827 | -1.074 | 0.048 |
| solute carrier family 22, member 17 | Slc22a17 | NM_177421 | -0.749 | 0.039 |
| solute carrier family 25, member 36 | Slc25a36 | CA511937 | -0.683 | 0.046 |
| solute carrier family 38, member 2 | Slc38a2 | NM_181090 | -0.600 | 0.042 |
| solute carrier family 4, sodium bicarbonate transporter-like, member 11 | Slc4a11 | AW917183 | -2.129 | 0.036 |
| SP100 nuclear antigen | Sp100 | AW915950 | -0.779 | 0.048 |
| SPARC related modular calcium binding 2 | Smoc2 | XM_214777 | -2.201 | 0.046 |
| sphingosine kinase 1 | Sphk1 | NM_133386 | -2.304 | 0.041 |
| spindle and kinetochore associated complex subunit 2 | Ska2 | NM_001009624 | -0.782 | 0.044 |
| spire homolog 1 (Drosophila) | Spire1 | CB545717 | -0.846 | 0.037 |
| splicing factor 3b, subunit 1 | Sf3b1 | XM_343570 | -0.594 | 0.044 |
| splicing factor 3b, subunit 2 | Sf3b2 | XM_215182 | -0.658 | 0.044 |
| splicing factor, arginine/serine rich 9 | Sfrs9 | NM_001009255 | -1.014 | 0.037 |
| src homology 2 domain-containing transforming protein D | Shd | XM_237255 | -0.663 | 0.047 |
| stonin 2 | Ston2 | XM_234454 | -0.605 | 0.042 |
| stromal antigen 1 | Stag1 | XM_236609 | -0.732 | 0.043 |
| structure specific recognition protein 1 | Ssrp1 | NM_031121 | -1.270 | 0.037 |
| suppressor of defective silencing 3 homolog (S. cerevisiae) | Suds3 | XM_341092 | -1.179 | 0.036 |
| suppressor of Ty 5 homolog (S. cerevisiae) | Supt5h | XM_218382 | -0.666 | 0.048 |
| sushi domain containing 3 | Susd3 | XM_225203 | -1.590 | 0.042 |
| SWAP-70 protein | Swap70 | XM_219262 | -1.405 | 0.036 |
| SWI/SNF related, matrix associated, actin dependent regulator of chromatin, subfamily a, member 5 | Smarca5 | XM_226380 | -0.724 | 0.042 |
| synaptojanin 2 | Synj2 | NM_032071 | -1.830 | 0.038 |
| synaptotagmin XI | Syt11 | NM_031667 | -0.970 | 0.047 |
| syndecan binding protein | Sdcbp | NM_031986 | -0.854 | 0.037 |
| syntaxin binding protein 5 (tomosyn) | Stxbp5 | NM_178345 | -1.121 | 0.043 |
| TAO kinase 3 | Taok3 | NM_001024254 | -0.697 | 0.044 |
| TATA box binding protein (TBP)-associated factor, RNA polymerase I, D, 41kDa | Taf1d | NM_001014207 | -0.975 | 0.036 |
| TBC1 domain family, member 17 | Tbc1d17 | XM_214940 | -0.730 | 0.044 |
| tetratricopeptide repeat domain 22 | Ttc22 | XM_233266 | -1.207 | 0.047 |
| tetratricopeptide repeat domain 39A | Ttc39a | XM_216483 | -2.169 | 0.044 |
| tetratricopeptide repeat domain 6 | Ttc6 | XM_216713 | -1.183 | 0.043 |
| TGFB-induced factor homeobox 1 | Tgif1 | NM_001015020 | -1.637 | 0.045 |
| thioredoxin domain containing 16 | Txndc16 | AW914923 | -0.949 | 0.04 |
| thyroid hormone receptor associated protein 3 | Thrap3 | AW141246 | -0.928 | 0.037 |
| thyroid hormone receptor interactor 4 | Trip4 | XM_236360 | -0.698 | 0.037 |
| toll-like receptor 2 | Tlr2 | NM_198769 | -1.748 | 0.04 |
| topoisomerase I binding, arginine/serine-rich | Topors | CB545427 | -1.062 | 0.037 |
| tousled-like kinase 1 | Tlk1 | XM_242032 | -0.674 | 0.047 |
| TRAF3 interacting protein 3 | Traf3ip3 | XM_341175 | -2.514 | 0.043 |
| transcription factor 19 | Tcf19 | BC093374 | -1.162 | 0.036 |
| transcription factor 20 | Tcf20 | XM_345861 | -0.846 | 0.04 |
| transducin-like enhancer of split 2 (E(sp1) homolog, Drosophila) | Tle2 | XM_216845 | -1.697 | 0.036 |
| transgelin | Tagln | NM_031549 | -1.788 | 0.037 |
| transmembrane and tetratricopeptide repeat containing 2 | Tmtc2 | XM_235136 | -0.674 | 0.046 |
| transmembrane protein 123 | Tmem123 | NM_001014205 | -0.830 | 0.046 |
| transmembrane protein 150C | Tmem150c | XM_341190 | -2.003 | 0.045 |
| transmembrane protein 176B | Tmem176b | NM_134390 | -0.750 | 0.043 |
| transporter 1, ATP-binding cassette, sub-family B (MDR/TAP) | Tap1 | NM_032055 | -1.335 | 0.049 |
| tripartite motif-containing 27 | Trim27 | XM_214485 | -0.606 | 0.045 |
| tripartite motif-containing 28 | Trim28 | XM_344861 | -1.374 | 0.04 |
| tripartite motif-containing 29 | Trim29 | XM_236207 | -0.868 | 0.037 |
| TSC22 domain family, member 4 | Tsc22d4 | BC079190 | -1.122 | 0.045 |
| tuberous sclerosis 2 | Tsc2 | NM_012680 | -0.618 | 0.042 |
| tubulin, alpha 4A | Tuba4a | NM_001007004 | -1.878 | 0.045 |
| tubulin, beta 2b | Tubb2b | NM_001013886 | -1.308 | 0.036 |
| tumor necrosis factor (ligand) superfamily, member 9 | Tnfsf9 | NM_181384 | -0.846 | 0.049 |
| tumor protein p53 inducible protein 11 | Tp53i11 | XM_230296 | -0.848 | 0.037 |
| tumor suppressing subtransferable candidate 4 | Tssc4 | NM_001013194 | -0.642 | 0.042 |
| TYRO3 protein tyrosine kinase | Tyro3 | NM_017092 | -0.950 | 0.048 |
| ubiquitin A-52 residue ribosomal protein fusion product 1 | Uba52 | NM_031687 | -0.728 | 0.039 |
| ubiquitin D | Ubd | NM_053299 | -2.066 | 0.037 |
| ubiquitin specific peptidase 18 | Usp18 | NM_001014058 | -1.099 | 0.037 |
| ubiquitin specific peptidase 28 | Usp28 | AA850333 | -0.841 | 0.037 |
| ubiquitin specific peptidase 3 | Usp3 | NM_001025424 | -1.048 | 0.036 |
| ubiquitin specific peptidase 31 | Usp31 | XM_219292 | -0.634 | 0.046 |
| ubiquitin specific protease 48 | Usp48 | NM_198785 | -0.942 | 0.036 |
| ubiquitin-conjugating enzyme E2A, RAD6 homolog (S. cerevisiae) | Ube2a | NM_001013933 | -0.820 | 0.046 |
| ubiquitin-conjugating enzyme E2I | Ube2i | CF110335 | -0.747 | 0.038 |
| ubiquitin-like modifier activating enzyme 6 | Uba6 | XM_223308 | -1.308 | 0.037 |
| ubiquitin-like with PHD and ring finger domains 2 | Uhrf2 | XM_219801 | -0.897 | 0.045 |
| UDP-Gal:betaGlcNAc beta 1,3-galactosyltransferase, polypeptide 4 | B3galt4 | NM_133553 | -0.614 | 0.048 |
| UDP-Gal:betaGlcNAc beta 1,4-galactosyltransferase, polypeptide 3 | B4galt3 | NM_001009539 | -0.812 | 0.037 |
| UNC-119 homolog (C. elegans) | Unc119 | NM_017188 | -1.426 | 0.036 |
| unc-13 homolog D (C. elegans) | Unc13d | NM_138844 | -1.612 | 0.036 |
| uncoupling protein 3 (mitochondrial, proton carrier) | Ucp3 | NM_013167 | -0.983 | 0.042 |
| UPF3 regulator of nonsense transcripts homolog B (yeast) | Upf3b | XM_233312 | -1.096 | 0.036 |
| UV radiation resistance associated gene | Uvrag | XM_218951 | -0.942 | 0.037 |
| vacuolar protein sorting 13 homolog A (S. cerevisiae) | Vps13a | XM_219672 | -1.150 | 0.038 |
| valyl-tRNA synthetase 2, mitochondrial (putative) | Vars2 | NM_213563 | -0.809 | 0.042 |
| vasodilator-stimulated phosphoprotein | Vasp | XM_341799 | -1.500 | 0.036 |
| vav 1 guanine nucleotide exchange factor | Vav1 | NM_012759 | -1.942 | 0.037 |
| vav 2 guanine nucleotide exchange factor | Vav2 | BF287543 | -1.182 | 0.036 |
| versican | Vcan | XM_215451 | -1.012 | 0.042 |
| von Willebrand factor A domain containing 1 | Vwa1 | NM_001013938 | -1.111 | 0.036 |
| Vpr (HIV-1) binding protein | Vprbp | XM_236612 | -0.875 | 0.036 |
| Vps20-associated 1 homolog (S. cerevisiae) | Vta1 | NM_001025640 | -0.932 | 0.049 |
| WD repeat domain 33 | Wdr33 | XM_226076 | -0.695 | 0.047 |
| WD repeat domain 70 | Wdr70 | NM_001013909 | -1.075 | 0.037 |
| WD repeat domain 74 | Wdr74 | XM_574613 | -0.939 | 0.037 |
| whey acidic protein | Wap | NM_053751 | -2.862 | 0.036 |
| WW domain containing E3 ubiquitin protein ligase 2 | Wwp2 | XM_214669 | -0.768 | 0.036 |
| Z-DNA binding protein 1 | Zbp1 | XM_342594 | -1.387 | 0.037 |
| zeta-chain (TCR) associated protein kinase | Zap70 | NM_001012002 | -2.567 | 0.046 |
| zinc finger CCCH type containing 7 A | Zc3h7a | XM_340742 | -1.040 | 0.036 |
| zinc finger protein 131 | Zfp131 | XM_227104 | -0.689 | 0.04 |
| zinc finger protein 394 | Znf394 | NM_145724 | -0.674 | 0.048 |
| zinc finger protein 46 | Zfp46 | AF151710 | -0.585 | 0.046 |
| zinc finger protein 653 | Zfp653 | XM_217093 | -0.638 | 0.047 |
| zinc finger protein ZFP | LOC503192 | XM_578715 | -0.664 | 0.044 |
| zinc finger RNA binding protein | Zfr | XM_345169 | -0.925 | 0.044 |
| zinc finger with UFM1-specific peptidase domain | Zufsp | NM_001008308 | -0.708 | 0.049 |
| zinc finger, CCHC domain containing 10 | Zcchc10 | XM_346887 | -0.808 | 0.037 |
| zinc finger, CCHC domain containing 7 | Zcchc7 | XM_216407 | -0.779 | 0.042 |
| zinc finger, FYVE domain containing 20 | Zfyve20 | XM_232195 | -0.727 | 0.046 |
