# Supplementary material for: In utero exposure to butyl benzyl phthalate induces modifications in the morphology and the gene expression profile of the mammary gland: an experimental study in rats
Source: Environ Health. 2011 Jan 17;10:5. doi: 10.1186/1476-069X-10-5 (PMC3033239; doi:10.1186/1476-069X-10-5)
Supplement: Additional file 3 — Functional categories analysis of the modulated genes by effect of in utero exposure to BBP. Detailed functional categories significantly over- or under-represented in the list of genes found as modulated at 35 days of age by the effects of in utero exposure to low (120 mg/kg BW) and high dose high dose (500 mg/kg BW) of BBP. [file 1476-069X-10-5-S3.DOC]

**Additional Table 3.**

Detailed functional categories significantly over- or under-represented in the list of genes found as modulated at 35 days of age by the effects of *in utero* exposure to low (120 mg/kg BW) and high dose high dose (500 mg/kg BW) of BBP.

|  | Function | GO | Number of genes in Rattus norvegicus database | Number of genes found in microarrays | Expected number of genes in microarrays | over (+) or under (-) represented | p-value |
| --- | --- | --- | --- | --- | --- | --- | --- |
| Low dose: |  |  |  |  |  |  |  |
| Up-modulated | signal transduction | GO:0007165 | 5088 | 12 | 4.58 | + | 6.86E-04 |
|  | cell communication | GO:0007154 | 5275 | 12 | 4.75 | + | 9.62E-04 |
|  | blood circulation | GO:0008015 | 241 | 3 | 0.22 | + | 1.30E-03 |
|  | muscle contraction | GO:0006936 | 544 | 4 | 0.49 | + | 1.34E-03 |
|  | metabolic process | GO:0008152 | 10671 | 17 | 9.61 | + | 2.64E-03 |
|  | apoptosis | GO:0006915 | 1185 | 5 | 1.07 | + | 3.68E-03 |
|  | primary metabolic process | GO:0044238 | 10150 | 16 | 9.14 | + | 4.87E-03 |
|  | intracellular signaling cascade | GO:0007242 | 1924 | 6 | 1.73 | + | 6.23E-03 |
|  | transport | GO:0006810 | 3282 | 8 | 2.96 | + | 6.42E-03 |
|  | cellular process | GO:0009987 | 7571 | 13 | 6.82 | + | 7.55E-03 |
| Down-modulated | primary metabolic process | GO:0044238 | 10150 | 111 | 77.52 | + | 2.00E-06 |
|  | metabolic process | GO:0008152 | 10671 | 113 | 81.5 | + | 8.10E-06 |
|  | nucleobase, nucleoside, nucleotide and nucleic acid metabolic process | GO:0006139 | 4537 | 59 | 34.65 | + | 1.79E-05 |
|  | cellular process | GO:0009987 | 7571 | 85 | 57.82 | + | 3.63E-05 |
|  | intracellular protein transport | GO:0006886 | 1859 | 30 | 14.2 | + | 9.09E-05 |
|  | protein transport | GO:0015031 | 1859 | 30 | 14.2 | + | 9.09E-05 |
|  | transport | GO:0006810 | 3282 | 44 | 25.07 | + | 1.44E-04 |
|  | endocytosis | GO:0006897 | 689 | 15 | 5.26 | + | 3.08E-04 |
|  | vesicle-mediated transport | GO:0016192 | 1337 | 22 | 10.21 | + | 6.48E-04 |
|  | carbohydrate metabolic process | GO:0005975 | 1186 | 20 | 9.06 | + | 8.52E-04 |
|  | cell cycle | GO:0007049 | 2233 | 30 | 17.05 | + | 1.84E-03 |
|  | immune system process | GO:0002376 | 3447 | 41 | 26.33 | + | 2.64E-03 |
|  | cell-matrix adhesion | GO:0007160 | 183 | 6 | 1.4 | + | 3.05E-03 |
|  | protein metabolic process | GO:0019538 | 4486 | 50 | 34.26 | + | 3.26E-03 |
|  | apoptosis | GO:0006915 | 1185 | 18 | 9.05 | + | 4.63E-03 |
|  | neurotransmitter secretion | GO:0007269 | 349 | 8 | 2.67 | + | 5.80E-03 |
|  | chromosome segregation | GO:0007059 | 280 | 7 | 2.14 | + | 6.14E-03 |
|  | mitosis | GO:0007067 | 770 | 13 | 5.88 | + | 6.73E-03 |
| High dose: |  |  |  |  |  |  |  |
| Up-modulated | metabolic process | GO:0008152 | 10671 | 192 | 125.32 | + | 6.71E-14 |
|  | primary metabolic process | GO:0044238 | 10150 | 176 | 119.21 | + | 1.17E-10 |
|  | cell communication | GO:0007154 | 5275 | 108 | 61.95 | + | 1.04E-09 |
|  | signal transduction | GO:0007165 | 5088 | 104 | 59.76 | + | 2.78E-09 |
|  | transport | GO:0006810 | 3282 | 76 | 38.54 | + | 5.14E-09 |
|  | immune system process | GO:0002376 | 3447 | 76 | 40.48 | + | 4.12E-08 |
|  | lipid metabolic process | GO:0006629 | 1365 | 40 | 16.03 | + | 1.35E-07 |
|  | cellular process | GO:0009987 | 7571 | 132 | 88.92 | + | 1.73E-07 |
|  | cell adhesion | GO:0007155 | 1543 | 42 | 18.12 | + | 4.50E-07 |
|  | system process | GO:0003008 | 2863 | 63 | 33.62 | + | 8.59E-07 |
|  | blood coagulation | GO:0007596 | 388 | 18 | 4.56 | + | 1.18E-06 |
|  | response to external stimulus | GO:0009605 | 388 | 18 | 4.56 | + | 1.18E-06 |
|  | carbohydrate metabolic process | GO:0005975 | 1186 | 34 | 13.93 | + | 2.04E-06 |
|  | neurological system process | GO:0050877 | 2537 | 55 | 29.8 | + | 7.54E-06 |
|  | cell-matrix adhesion | GO:0007160 | 183 | 11 | 2.15 | + | 1.43E-05 |
|  | intracellular protein transport | GO:0006886 | 1859 | 43 | 21.83 | + | 1.92E-05 |
|  | protein transport | GO:0015031 | 1859 | 43 | 21.83 | + | 1.92E-05 |
|  | intracellular signaling cascade | GO:0007242 | 1924 | 44 | 22.6 | + | 2.00E-05 |
|  | protein metabolic process | GO:0019538 | 4486 | 82 | 52.69 | + | 2.13E-05 |
|  | oxygen and reactive oxygen species metabolic process | GO:0006800 | 70 | 7 | 0.82 | + | 2.34E-05 |
|  | endocytosis | GO:0006897 | 689 | 22 | 8.09 | + | 2.93E-05 |
|  | response to stimulus | GO:0050896 | 2759 | 56 | 32.4 | + | 3.96E-05 |
|  | lipid transport | GO:0006869 | 296 | 13 | 3.48 | + | 6.23E-05 |
|  | homeostatic process | GO:0042592 | 157 | 9 | 1.84 | + | 1.22E-04 |
|  | cell-cell adhesion | GO:0006886 | 957 | 25 | 11.24 | + | 2.02E-04 |
|  | cell surface receptor linked signal transduction | GO:0007166 | 2732 | 53 | 32.09 | + | 2.05E-04 |
|  | nitrogen compound metabolic process | GO:0006807 | 74 | 6 | 0.87 | + | 2.76E-04 |
|  | vesicle-mediated transport | GO:0016192 | 1337 | 31 | 15.7 | + | 2.88E-04 |
|  | cellular amino acid and derivative metabolic process | GO:0006519 | 411 | 14 | 4.83 | + | 4.41E-04 |
|  | developmental process | GO:0032502 | 3611 | 64 | 42.41 | + | 5.03E-04 |
|  | synaptic transmission | GO:0007268 | 636 | 18 | 7.47 | + | 6.38E-04 |
|  | blood circulation | GO:0008015 | 241 | 10 | 2.83 | + | 6.66E-04 |
|  | cell-cell signaling | GO:0007267 | 1420 | 30 | 16.68 | + | 1.55E-03 |
|  | cellular glucose homeostasis | GO:0001678 | 72 | 5 | 0.85 | + | 1.76E-03 |
|  | apoptosis | GO:0006915 | 1185 | 26 | 13.92 | + | 1.91E-03 |
|  | anterior/posterior axis specification | GO:0009948 | 109 | 6 | 1.28 | + | 2.01E-03 |
|  | coenzyme metabolic process | GO:0006732 | 109 | 6 | 1.28 | + | 2.01E-03 |
|  | nervous system development | GO:0007399 | 1480 | 30 | 17.38 | + | 2.86E-03 |
|  | generation of precursor metabolites and energy | GO:0006091 | 619 | 16 | 7.27 | + | 3.07E-03 |
|  | response to stress | GO:0006950 | 681 | 17 | 8 | + | 3.28E-03 |
|  | system development | GO:0048731 | 2392 | 43 | 28.09 | + | 3.61E-03 |
|  | visual perception | GO:0007601 | 472 | 13 | 5.54 | + | 4.37E-03 |
|  | ectoderm development | GO:0007398 | 1678 | 32 | 19.71 | + | 5.16E-03 |
|  | vitamin metabolic process | GO:0006766 | 93 | 5 | 1.09 | + | 5.18E-03 |
|  | response to toxin | GO:0009636 | 133 | 6 | 1.56 | + | 5.26E-03 |
|  | ion transport | GO:0006811 | 838 | 19 | 9.84 | + | 5.39E-03 |
|  | sulfur metabolic process | GO:0006790 | 135 | 6 | 1.59 | + | 5.65E-03 |
|  | vitamin transport | GO:0051180 | 107 | 5 | 1.26 | + | 9.16E-03 |
|  | carbohydrate transport | GO:0008643 | 197 | 7 | 2.31 | + | 9.37E-03 |
|  | cation transport | GO:0006812 | 700 | 16 | 8.22 | + | 9.51E-03 |
|  | respiratory electron transport chain | GO:0022904 | 581 | 14 | 6.82 | + | 9.71E-03 |
| Down-modulated | primary metabolic process | GO:0044238 | 10150 | 295 | 191.61 | + | 3.71E-20 |
|  | cellular process | GO:0009987 | 7571 | 235 | 142.92 | + | 5.88E-18 |
|  | metabolic process | GO:0008152 | 10671 | 298 | 201.44 | + | 1.14E-17 |
|  | nucleobase, nucleoside, nucleotide and nucleic acid metabolic process | GO:0006139 | 4537 | 154 | 85.65 | + | 7.11E-14 |
|  | cell cycle | GO:0007049 | 2233 | 93 | 42.15 | + | 6.21E-13 |
|  | protein metabolic process | GO:0019538 | 4486 | 142 | 84.68 | + | 1.65E-10 |
|  | cell communication | GO:0007154 | 5275 | 156 | 99.58 | + | 2.02E-09 |
|  | intracellular signaling cascade | GO:0007242 | 1924 | 73 | 36.32 | + | 1.39E-08 |
|  | signal transduction | GO:0007165 | 5088 | 145 | 96.05 | + | 1.05E-07 |
|  | cell motion | GO:0006928 | 1039 | 44 | 19.61 | + | 8.25E-07 |
|  | transport | GO:0006810 | 3282 | 97 | 61.96 | + | 5.83E-06 |
|  | cell-cell signaling | GO:0007267 | 1420 | 51 | 26.81 | + | 1.14E-05 |
|  | establishment or maintenance of chromatin architecture | GO:0006325 | 477 | 24 | 9.00 | + | 2.00E-05 |
|  | cellular component organization | GO:0016043 | 1907 | 62 | 36.00 | + | 2.56E-05 |
|  | carbohydrate transport | GO:0008643 | 197 | 14 | 3.72 | + | 3.24E-05 |
|  | organelle organization | GO:0006996 | 516 | 24 | 9.74 | + | 6.72E-05 |
|  | intracellular protein transport | GO:0006886 | 1859 | 59 | 35.09 | + | 7.85E-05 |
|  | protein transport | GO:0015031 | 1859 | 59 | 35.09 | + | 7.85E-05 |
|  | apoptosis | GO:0006915 | 1185 | 42 | 22.37 | + | 9.20E-05 |
|  | immune system process | GO:0002376 | 3447 | 95 | 65.07 | + | 1.07E-04 |
|  | cell-cell adhesion | GO:0016337 | 957 | 35 | 18.07 | + | 1.99E-04 |
|  | system development | GO:0048731 | 2392 | 69 | 45.15 | + | 3.14E-04 |
|  | carbohydrate metabolic process | GO:0005975 | 1186 | 40 | 22.39 | + | 3.64E-04 |
|  | mitosis | GO:0007067 | 770 | 29 | 14.54 | + | 4.36E-04 |
|  | neurotransmitter secretion | GO:0007269 | 349 | 17 | 6.59 | + | 4.51E-04 |
|  | negative regulation of apoptosis | GO:0043066 | 321 | 16 | 6.06 | + | 5.16E-04 |
|  | developmental process | GO:0032502 | 3611 | 95 | 68.17 | + | 5.31E-04 |
|  | cell adhesion | GO:0007155 | 1543 | 48 | 29.13 | + | 5.75E-04 |
|  | generation of precursor metabolites and energy | GO:0006091 | 619 | 2 | 11.69 | - | 6.21E-04 |
|  | response to stress | GO:0006950 | 681 | 26 | 12.86 | + | 7.00E-04 |
|  | cell surface receptor linked signal transduction | GO:0007166 | 2732 | 74 | 51.57 | + | 1.10E-03 |
|  | respiratory electron transport chain | GO:0022904 | 581 | 2 | 10.97 | - | 1.15E-03 |
|  | neurological system process | GO:0050877 | 2537 | 69 | 47.89 | + | 1.48E-03 |
|  | response to stimulus | GO:0050896 | 2759 | 73 | 52.08 | + | 2.18E-03 |
|  | nervous system development | GO:0007399 | 1480 | 44 | 27.94 | + | 2.29E-03 |
|  | cytokinesis | GO:0000910 | 271 | 13 | 5.12 | + | 2.32E-03 |
|  | system process | GO:0003008 | 2863 | 75 | 54.05 | + | 2.45E-03 |
|  | visual perception | GO:0007601 | 472 | 18 | 8.91 | + | 4.46E-03 |
|  | vesicle-mediated transport | GO:0016192 | 1337 | 39 | 25.24 | + | 5.42E-03 |
|  | synaptic transmission | GO:0007268 | 636 | 22 | 12.01 | + | 5.54E-03 |
|  | gamete generation | GO:0007276 | 1023 | 31 | 19.31 | + | 7.53E-03 |
|  | ectoderm development | GO:0007398 | 1678 | 46 | 31.68 | + | 7.92E-03 |
|  | cell-matrix adhesion | GO:0007160 | 183 | 9 | 3.45 | + | 8.89E-03 |
